# Dissecting the Kinetic Mechanism of Human Lysine Methyltransferase 2D and Its Interactions with the WRAD2 Complex

**DOI:** 10.1021/acs.biochem.2c00385

**Published:** 2022-09-07

**Authors:** Lucy V. Edwardes, Sarah J. Caswell, Mariacarmela Giurrandino, Xiang Zhai, Andrea Gohlke, Demetrios H. Kostomiris, Hannah K. Pollard, Alexander Pflug, Gregory R. Hamm, Kate V. Jervis, Paul N. Clarkson, Karl Syson

**Affiliations:** † Discovery Biology, Discovery Sciences, BioPharmaceuticals, R&D, AstraZeneca, Cambridge CB4 0WG, U.K.; ‡ Mechanistic and Structural Biology, Discovery Sciences, BioPharmaceuticals, R&D, AstraZeneca, Boston, Massachusetts 02210, United States; § Mechanistic and Structural Biology, Discovery Sciences, BioPharmaceuticals, R&D, AstraZeneca, Cambridge CB4 0WG, U.K.; ∥ Imaging and Data Analytics, Clinical Pharmacology and Safety Sciences, BioPharmaceuticals R&D, AstraZeneca, Cambridge CB4 0WG, U.K.

## Abstract

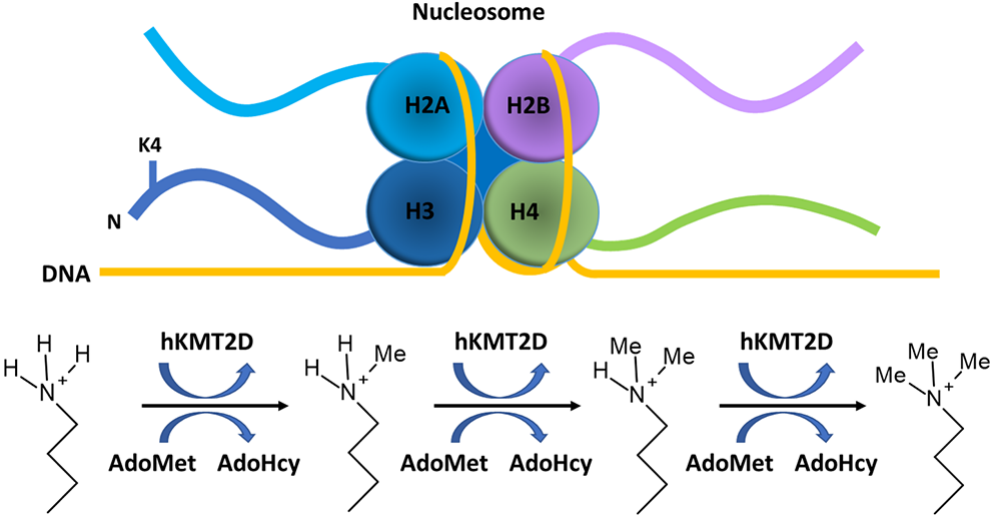

Human lysine methyltransferase 2D (hKMT2D) is an epigenetic writer catalyzing the methylation of histone 3 lysine 4. hKMT2D by itself has little catalytic activity and reaches full activation as part of the WRAD2 complex, additionally comprising binding partners WDR5, RbBP5, Ash2L, and DPY30. Here, a detailed mechanistic study of the hKMT2D SET domain and its WRAD2 interactions is described. We characterized the WRAD2 subcomplexes containing full-length components and the hKMT2D SET domain. By performing steady-state analysis as a function of WRAD2 concentration, we identified the inner stoichiometry and determined the binding affinities for complex formation. Ash2L and RbBP5 were identified as the binding partners critical for the full catalytic activity of the SET domain. Contrary to a previous report, product and dead-end inhibitor studies identified hKMT2D as a rapid equilibrium random Bi–Bi mechanism with EAP and EBQ dead-end complexes. Matrix-assisted laser desorption ionization time-of-flight mass spectrometry (MALDI-ToF MS) analysis showed that hKMT2D uses a distributive mechanism and gives further insights into how the WRAD2 components affect mono-, di-, and trimethylation. We also conclude that the Win motif of hKMT2D is not essential in complex formation, unlike other hKMT2 proteins.

## Introduction

Epigenetic control is mediated by enzymatic introduction or removal of covalent modifications to histone proteins or by directly modifying DNA and RNA through chromatin remodeling. Histones are small alkaline proteins with unstructured N-terminal tails that are prone to post-translational modifications (PTMs).^1−3^ Said modifications include phosphorylation, methylation, acetylation, ubiquitination, and SUMOylation of residue side chains such as lysine, arginine, histidine, and serines also referred to as the “histone code.”^4,5^ The coordinated deposition, interpretation, and removal of these PTMs, required to achieve the correct biological effect, are profoundly complex, and the interplay between histone code readers and writers is still not completely understood.^6^ Histone tail PTMs can confer control over gene transcription either directly through promoting binding of transcription factors or indirectly through mediating chromatin structure reorganization, altering DNA accessibility.^3,7,8^

Human lysine methyltransferases (hKMTs) are a superfamily that can be divided into five classes which transfer methyl groups from the methyl donor *S*-adenosyl-l-methionine (AdoMet) to the ε-amino group of lysines, producing *S*-adenosyl-l-homocysteine (AdoHcy) as a byproduct.^5,9−11^ In mammalian cells, methylation of DNA, histones, and other proteins is as common as phosphorylation and ubiquitination.^12^ Unlike other PTMs that are mainly recognized by charge or size differences, such as phosphorylation and ubiquitination, respectively, the addition of 1, 2, or 3 methyl groups does not alter the overall charge of the ε-amino group of lysine at neutral pH and only contributes a modest 14 Da to the overall protein.^13^ Lysine side chains are commonly involved in salt bridge or hydrogen bond formation; however, as the methylation state of a lysine side chain increases, the hydrogen bond potential decreases. Conversely, the addition of a methyl can create an unconventional CH–O hydrogen bond;^14−17^ therefore, effector proteins that recognize different intermediate states of methyllysine must be fine-tuned to discriminate between different methylation states.

In humans, class I and V methyltransferases act on histones and differ, respectively, by the absence or presence of a catalytic SET domain.^11^ The SET (SU(var), Enhancer of Zeste and Trithorax) domain is formed by ∼140 residues, highly conserved in its sequence, and present in all studied eukaryotes.^1,10,18^ The class V methyltransferases are further subdivided into seven known SET families: SUV3/9, SET1, SET2, EZ, RIZ, SMYD, and SUV4–20.^10^

Common with many proteins involved in epigenetic control, the SET1/MLL/KMT2 family of methyltransferases is of therapeutic interest as dysregulation or mutation has been found to be involved in various cancers, frequently with mutations located in the catalytic SET domain.^19−22^ The KMT2 methyltransferases are again divided into subgroups based on their sequence homology and methylation activity.^1,23^ hKMT2A/B (MLL1/2) show homology with *Drosophila melanogaster* trithorax (Trx) and primarily regulate Hox genes through trimethylation, whereas hKMT2F/G (MLL5/6 or SET1A/B) trimethylate at promoter regions and show homology to the Set1 protein of both *Saccharomyces cerevisiae* and *D. melanogaster*. hKMT2C/D (MLL3/4) share their sequence homology with *D. melanogaster* trithorax-related protein (Trr) and preferentially monomethylate enhancer regions of actively transcribed genes.^19,24−27^ Monomethylation at enhancer regions is implicated in the accessibility and activation of these regions, and methylation performed by hKMT2D has been observed as necessary for recruitment and activation of FOXA1, PBX1, and ER α TF to specific chromatin sites.^19,27,28^ hKMT2s are large proteins ranging from 1707 to 5537 residues, with the isolated proteins having little activity unless associated with the WRAD2 complex.^1,2,19,29,30^ The WRAD2 complex consists of four proteins, WDR5 (WD repeat domain), RbBP5 (retinoblastoma-binding protein), ASH2L (absent small or homoeotic 2-like), and homodimer DPY30 (Dumpy-30).^2,31^ It is thought that forming the hKMT2:WRAD2 complex alters the active site conformation, allowing optimum alignment of the methyl donor and acceptor for an efficient S_N_2 reaction.^32^ The WDR5 interacting motif (Win motif) of the Win-SET domain is also thought to be essential in WRAD2 complex formation in hKMT2 enzymes and is driven by the critical initial formation of the Win–WDR5 interaction via a conserved Win motif arginine residue.^29,32,33^

Given the size of these proteins, hKMT2D is the largest of the family at 5537 amino acids,^1^ and most in vitro studies have used truncated constructs focusing on the Win-SET region for both functional and structural studies.^29,32−35^ Understanding an enzyme’s catalytic mechanism is important, as during the catalytic cycle, the enzyme presents numerous intermediates through the binding of substrates and formation of products.^36^ A number of publications have reported hKMT2D kinetic parameters and the effect of the WRAD2 complex on catalysis, but few have performed full mechanistic analysis, with one group reporting a sequential Bi–Bi mechanism.^37^ Here, we expressed the hKMT2D SET domain and the individual WRAD2 proteins. Measurement of the steady state and product and dead-end inhibitor parameters identifies the hKMT2D mechanism as a rapid equilibrium random Bi–Bi mechanism with EAP and EBQ dead-end complexes. Monitoring products over time with matrix-assisted laser desorption ionization time-of-flight (MALDI-ToF) mass spectrometry shows that hKMT2D uses a distributive enzyme mechanism with monomethylation being the most efficient reaction. Furthermore, we identify the key interactions of the WRAD2 complex and a minimal complex that processes activity that is equivalent to that of the full WRAD2 complex.

## Materials and Methods

### Reagents

The following peptides were all purchased from Chinese Peptide Company. H3 peptides were derived from the first 21 amino acids of human H3 histone with the sequence ARTKQTARKSTGGKAPRKQLA. All peptides used were modified at the lysine four position and nonacetylated at the N-terminus. H3 histone peptide (H3_1–21_); monomethylated H3_1–21_ (Me1H3_1–21_), dimethylated H3_1–21_ (Me2H3_1–21_), and trimethylated histone H3_1–21_ (Me3H3_1–21_); norleucine H3_1–21_ (NleH3_1–21_); and a 34 amino acid RbBP5 peptide SAFAPDFKELDENVEYEERESEFDIEDEDKSEPE corresponded to residues 330 to 363. HeLa oligonucleosomes were purchased from Reaction Biology Corporation. H3.1K4me0, H3.1K4me1, and H3.1K4me3 recombinant mononucleosomes were all purchased from Active Motif. MTase-Glo custom assay kits were purchased from Promega and contained *S*-adenosyl-l-homocysteine (AdoHcy), *S*-adenosyl methionine (AdoMet), methyltransferase-Glo reagent, and methyltransferase-Glo detection solution. α-Cyano-4-hydroxycinnamic acid (CHCA), *n*-dodecyl β-d-maltoside, Triton X-100, dithiothreitol (DTT), formic acid, dimethyl sulphoxide (DMSO), trifluoracetic acid (TFA), sodium chloride (NaCl), imidazole, Tris(2-carboxyethyl)phosphine hydrochloride (TCEP), glycerol, and tris(hydroxymethyl)aminomethane (Tris) were all purchased from Sigma-Aldrich. Assays were run in Greiner 384 well low volume plates (784,075). Size exclusion and nickel affinity columns were purchased from GE Healthcare.

### Expression and Purification of Human KMT2D and WRAD2 Components

DNA sequence coding for variants of human WDR5, RbBP5, ASH2L, and DPY30 constructs were cloned into a pET24a vector using golden gate assembly to produce the N-terminal 6His-tag fusion protein with a tobacco etch virus (TEV) protease site (Figure S1). Constructs were expressed in *Escherichia coli* Rosetta 2 (DE3). Bacteria were grown in Luria broth at 37 °C with shaking, induced at *A*_600_ = 0.5 with 0.1 mM IPTG, and incubated for 20 h at 18 °C. KMT2D SET and Win-SET proteins were expressed in Sf21 cells using a pFASTBAC vector and the Bac-2-Bac expression system.^38^ Cells were harvested by centrifugation and resuspended in five times volume per gram of cell pellet using lysis buffer (50 mM Tris–HCl pH 7.4, 300 mM NaCl, 10% glycerol, 1 mM TCEP, 20 mM imidazole, 1× EDTA-free mini complete protease inhibitors (Roche) per 50 mL and 0.1 U/mL benzonase) and lysed using a Constant Systems cell disruptor at 30 Kpsi. The lysate was cleared by centrifugation at 48,000*g* for 2 h at 4 °C and then applied to a 5 mL HisTrap FF Ni^2+^ Sepharose metal ion affinity chromatography column. This was followed by 50 CV of wash buffer (50 mM Tris–HCl pH 7.4, 300 mM NaCl, 10% glycerol, 1 mM TCEP, 20 mM imidazole) at 4 °C. Bound proteins were eluted from the column using a step gradient using 10 CV of wash buffer containing 300 mM imidazole. The protein was dialyzed for 20 h against 4 L of dialysis buffer (50 mM Tris–HCl pH 7.4, 300 mM NaCl, 10% glycerol, 1 mM TCEP), plus 1:20 6His–TEV protease to the target protein. His-tagged TEV protease and free 6×His tag were removed by incubation of the eluent with 500 μL of Ni^2+^ Sepharose. After centrifugation, the supernatant was concentrated to 5 mL and applied to a Superdex 200 16/60 size exclusion column equilibrated with dialysis buffer. Complexes were reconstituted by incubating equimolar amounts of required proteins on ice for 1 h, and complexes were separated using a Superdex 200 26/60 size exclusion column equilibrated with dialysis buffer. Peak fractions were concentrated to approximately 20 mg/mL, flash-frozen in liquid nitrogen, and stored at −80 °C. Intact mass spectrometry was performed using a Sciex X500B Q-TOF with Sciex Excion LC instrument and a bioZen 3.6 μm Intact XB-C8 column. All proteins were diluted at least 10× in mass spec buffer (5% acetonitrile, 0.1% formic acid) to 0.1 mg/mL.

### Methyltransferase Luminescence Assay

SET domain activity was monitored with a quantitative endpoint assay determining AdoHcy production using MTase-Glo by Promega.^39^ Assays were performed as time courses at room temperature with buffer constituents, 50 mM Tris, pH 7.5, 50 mM NaCl, 1 mM DTT, 1% DMSO, and 0.005% w/v Triton X-100 in deionized water. The SET domain was incubated with substrates AdoMet and H3_1–21_ peptide, in a final volume of 4 μL. Addition of 1 μL of 0.5% v/v TFA was used to stop the methylation reaction at defined time points; 1 μL of 6× concentrated MTase-Glo reagent was added to each well and incubated at room temperature. After 30 min, 6 μL of the prefiltered MTase-Glo detection reagent was added and incubated for a further 30 min at room temperature. Luminescence was measured using an Envision 2101 Multilabel plate reader, and product concentrations were calculated using an AdoHcy standard curve. Steady-state rates were obtained by plotting AdoHcy production over time and normalized to SET domain concentration. Experiments were performed in triplicate and expressed as the mean ± SD. Data were analyzed using nonlinear regression in GraphPad Prism v9.1.

### Steady-State Studies

Steady-state rates were measured in substrate matrix experiments. Data were globally fitted to ternary Bi–Bi and Ping–Pong models to obtain *k*_cat_ and *K*_M_ parameters (eqs 1 and 2).

The ternary Bi–Bi model
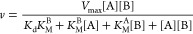
1The Ping–Pong model
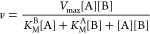
2where ν is the initial rate, *V*_max_ is the maximum velocity, [A] is the concentration of the varied substrate, [B] the concentration of the fixed substrate, *K*_M_^A^ and *K*_M_^B^ are the Michaelis constants of the varied and fixed substrates, respectively, and *K*_d_ is the dissociation constant of the varied substrate. A detailed WRAD2 titration was performed using SET, WRAD2, AdoMet, and H3_1–21_ peptide concentrations described in Figure S3 and fitted to eq 1.

The change in the catalytic parameters as a function of WRAD2 concentration was fitted to eq 3
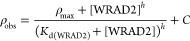
3where ρ_obs_ is the observed value of either *k*_cat_, 1/*K*_M_, or *k*_cat_/*K*_M_, ρ_max_ is the maximal value of either *k*_cat_, 1/*K*_M_, or *k*_cat_/*K*_M_, [WRAD2] is the concentration of the WRAD2 complex, *h* is the Hill coefficient, and *C* is the basal activity of the SET domain in the absence of WRAD2.

The change in H3_1–21_ binding to the free enzyme was fitted to eq 4
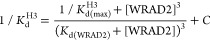
41/*K*_d_^H3^ is the reciprocal of the dissociation constant for H3_1–21_, 1/*K*_d(max)_^H3^ is the maximal value of the reciprocal of the dissociation constant for H3_1–21_, [WRAD2] is the concentration of the WRAD2 complex, and *C* is the background measurement.

Me1H3_1–21_ and Me2H3_1–21_ substrate matrix experiments used 10 or 50 nM SET/WRAD2 in a 1:1 ratio, respectively. Substrate ranges for AdoMet and methylated H3_1–21_ were 0–50 and 0–500 μM respectively and fitted to eq 1. Recombinant mononucleosome titrations used 1:1 SET/WRAD2 concentrations of 11, 181, and 150 nM and AdoMet fixed at 20 μM. HeLa oligonucleosome titration used 13.5 nM SET/WRAD2 and AdoMet fixed at 20 μM. Data were fitted to the Michaelis–Menten equation
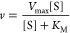
5where ν is the initial rate, *V*_max_ is the maximum velocity, [S] is the concentration of the varied substrate, and *K*_M_ is the Michaelis constant. The minimal complex matrix experiment using 20 nM SET/Ash2L/RbBP5 in a 1:1:1 ratio used a truncated Ash2L peptide (380–496-ISGRGS-539–598) and a 34 mer RbBP5 peptide (330–363) with 5, 10, 15, 20, 30, and 40 min time points. Substrate ranges for AdoMet and H3_1–21_ were 0–50 and 0–400 μM, respectively, and fitted to eq 1.

### Effect of Individual WRAD2 Components on SET Activity

Individual WRAD2 components, WDR5, RbBP5, Ash2L, and DPY30, were tested against the SET domain in a 1:1 ratio at 30 or 100 nM with 5 μM AdoMet with either 0–250 or 0–1000 μM H3_1–21_ peptide. Dpy30 was added in a 2:1 ratio. Data were fitted to eq 5.

### Dead-End and Product Inhibitor Studies

AdoHcy and the trimethylated Me3H3_1–21_ peptide were used as product inhibitors, while dead-end substrate analogues were sinefungin and NleH3_1–21_ peptide. Steady-state rates were measured in substrate-inhibitor matrix experiments. Dead-end inhibitor experiments were performed with the second substrate fixed at *K*_M_, while product inhibitor experiments fixed the second substrate at *K*_M_ or 20× *K*_M_. Assays using 20× *K*_M_ AdoMet used a cofactor adjusted to pH 7.5. AdoHcy inhibition experiments used a maximum concentration of 8 μM with optimized MTase-Glo additions of 1 μL of 10× MTase-Glo reagent and 12 μL of the MTase-Glo detection reagent, and reactions were monitored over 30 or 50 min with a 30 or 40 nM 1:1 SET–WRAD2 complex. Varied substrate concentrations of 200, 100, 50, 25, 12.5, 6.25, 3.125, and 1.5625 μM H3_1–21_ and 50, 25, 12.5 6.25, 3.125, 1.56, and 0.78 μM AdoMet were used for Me3H3_1–21_ and AdoHcy inhibition studies, respectively. Steady-state rates were globally fitted to competitive, uncompetitive, and noncompetitive inhibition (eqs 6, 7, and 8, respectively).

Competitive inhibition
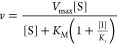
6Uncompetitive inhibition
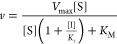
7Noncompetitive inhibition
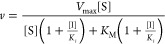
8Mixed inhibition
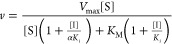
9where ν is the initial rate, *V*_max_ is the maximum velocity of the uninhibited reaction, [S] is the substrate concentration of the varied substrate, [I] is the inhibitor concentration, *K*_M_ is the Michaelis–Menten constant, and *K*_*i*_ is the inhibition constant. To resolve any ambiguities in assigning inhibition type, the mixed inhibition model was used (eq 9), to derive the value of α, which is a measure of competitive or uncompetitive nature.

### MALDI-ToF MS Time Course

Matrix-assisted laser desorption ionization time-of-flight mass spectrometry (MALDI-ToF MS) assays used 500 nM SET, 1:1 SET/Ash2L, 1:1 SET/BbBP5, 200 nM 1:1:1 SET/RbBP5/Ash2L, 1:1:1:1 SET/RbBP5/Ash2L/WDR5, 1:1:1:2 SET/RbBP5/Ash2L/DPY30, and 1:1:1:1:2 SET/RbBP5/Ash2L/WDR5/DPY30 with 200 μM AdoMet adjusted to pH 7.5 and 20 μM H3_1–21_. A minimal buffer system of 5 mM Tris, pH 7.5, 1 mM DTT, and 0.005% w/v *n*-dodecyl β-d-maltoside was used to avoid ion suppression of the species of interest; 5 μL of reaction aliquots were stopped at 0, 5, 10, 20, 30, 60, 120, 180, 240, 300, 360, 420, 480, and 1440 min time points with an equal volume of 0.2% v/v TFA. The samples were spotted onto a stainless steel MALDI target plate at 1 μL and then covered with 1 μL of the α-cyano-4-hydroxycinnamic acid (CHCA) matrix at 10 mg/mL, prepared in a 1:1 acetonitrile–water solution and allowed to dry at room temperature. MALDI-ToF MS experiments were performed on a Rapiflex TissueTyper (Bruker Daltonics, Bremen, Germany). All resulting spots were analyzed using the imaging mode. Images were collected at a spatial resolution of 200 μm in the positive detection mode over a mass range of 1000–3000 Da. Spectra were obtained by accumulating 600 laser shots per pixel with a frequency of 10 kHz. The laser beam diameter was adjusted at 50 μm. FlexControl 5.0 and FlexImaging 5.0 (Bruker Daltonics) were used for MS parameter optimization and MSI experiment setup, respectively. Mean spectra were extracted for each spot as.csv files using SCiLS Lab MVS 2020a software (SCiLS GmbH, Bremen, Germany), and the peak integrations were calculated to determine the concentration of each product using eq 10, compensating for spot-to-spot variations.
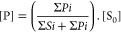
10where [P] is the concentration of the product, ∑*Pi* is the sum of the product peak integrals, ∑*Si* is the sum of the substrate peak integrals, and [S_0_] is the starting concentration of the substrate. Progress curves were fitted to sequential methylation models for two or three methylations using KinTek Explorer v10.^40^
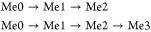
where Me0, Me1, Me2, and Me3 correspond to non-, mono-, di-, and trimethylated H3_1–21_ peptides, respectively.

### SPR Binding Assays

Surface plasmon resonance experiments were performed using a T200 instrument (Cytiva) equipped with a research-grade NTA sensor S chip (Cytiva) at 20 °C. For immobilization, the instrument was primed with a buffer composed of 10 mM HEPES, pH 7.5, 150 mM NaCl, 0.5 mM TCEP, and 0.05% Tween 20. The NTA chip was conditioned with three 2 min injections of 50 mM NaOH/1 M NaCl and a 2 min injection of 250 mM EDTA. Protein and reference flow cells were then prepared by a 2 min injection of 1 mM NiCl_2_ and a 7 min injection of 0.2 M EDC/ 0.05 M NHS. Immediately, the protein (500 nM His-SET (5382–5537) + ASH2L (380-496-ISGRGS-539-598) + RbBP5 peptide in immobilization buffer) was injected over the measurement flow cell to the desired RU level, followed by deactivation with a 7 min injection of 1 M ethanolamine pH 8 of all flow cells. For binding measurements, the system was then primed in a running buffer consisting of 50 mM Tris pH 7.5, 150 mM NaCl, 1 mM TCEP, 1–2% DMSO, and 0.05% Tween 20. Steady-state affinity data were recorded with NleH3_1–21_ and AdoMet prepared in running buffer and injected at a flow rate of 30 μL/min in a concentration-dependent manner over both protein and reference cells and recorded at 10 Hz. Data processing included solvent correction and blank subtraction. The steady-state data were analyzed using Biaevaluation/Insight Software 1.1 (GE Healthcare/Cytiva) using an implemented 1:1 interaction model.

## Results

### Protein Expression and Intact Mass Spectrometry

hKMT2D Win-SET was expressed in Sf21 cells, while WRAD2 proteins were expressed in *E. coli* and purified to homogeneity using column chromatography. Individual proteins were subjected to intact mass spectrometry to confirm the correct molecular mass. A list of amino acid sequences and tags can be seen in the Supporting Information (Figure S1). Intact mass spectrometry of the Win-SET protein showed a smaller than expected mass of 20,794 Da, differing from the expected mass of 29,266 Da (Figure S2). A loss of 8472 Da corresponds to the loss of the N-terminal 6×His tag, TEV cleavage site, and amino acids 5308 to 5361 including the Win motif. The conserved arginine at amino acid position 5340 is also within this cleaved region, a residue thought to be essential in Win-SET complex formation with WRAD2.^33^ It is most likely that the cleavage occurs after purification, as the initial purification step uses nickel column affinity. Efforts to express a nontruncated form of the Win-SET domain, by making point mutations around the amino acid 5360 cleavage site, were unsuccessful (data not shown). As the Win motif has been lost due to proteolysis, we shall refer to the catalytic subunit expressed here as the SET domain.

### WRAD2 Titration

To assess the effect of the WRAD2 complex on the catalytic parameters of the SET domain, a detailed WRAD2 titration was carried out using substrate matrix experiments by varying one substrate at a range of fixed concentrations of the second substrate. The data were globally fitted to eq 1 using nonlinear regression to determine *k*_cat_, *K*_M_ (AdoMet), *K*_M_ (H3_1–21_), and *K*_d_ (AdoMet) when AdoMet is the varied substrate and *K*_M_ (AdoMet), *K*_M_ (H3_1–21_), and *K*_d_ (H3_1–21_) when H3_1–21_ is varied (Table 1).

**Table 1:** Effect of WRAD2 Complex Concentration on SET Domain Steady-State Parameters^a^

[WRAD2] nM	*K*_M_^(AdoMet)^ (μM)	*K*_d_^(AdoMet)^ (μM)	*K*_M_^(H3)^ (μM)	*K*_d_^(H3)^ (μM)	*k*_cat_ (s^–1^)
0	5.38 ± 1.0	5.93 ± 1.20	512.70 ± 58.85	565.50 ± 161.00	0.0124 ± 0.0007
0.125	5.48 ± 1.25	4.99 ± 1.50	454.60 ± 69.70	413.80 ± 161.40	0.0119 ± 0.0008
0.25	6.28 ± 0.65	5.02 ± 0.68	503.90 ± 35.52	403.20 ± 70.30	0.0120 ± 0.0004
0.5	3.61 ± 0.51	2.93 ± 0.60	454.60 ± 39.92	368.40 ± 97.62	0.0093 ± 0.0004
1	4.55 ± 0.62	1.61 ± 0.58	277.90 ± 29.28	98.30 ± 39.45	0.0074 ± 0.0004
2	4.18 ± 0.50	0.61 ± 0.36	108.00 ± 11.38	13.69 ± 11.38	0.0085 ± 0.0003
4	2.36 ± 0.28	0.65 ± 0.38	36.93 ± 3.55	6.12 ± 5.51	0.0074 ± 0.0003
6.25	3.07 ± 0.16	1.54 ± 0.36	17.73 ± 0.92	8.69 ± 2.06	0.0295 ± 0.0004
10	3.89 ± 0.33	1.42 ± 0.56	18.25 ± 1.49	6.67 ± 2.64	0.0669 ± 0.0017
15	4.77 ± 0.22	0.99 ± 0.30	18.21 ± 0.84	3.76 ± 1.14	0.0952 ± 0.0015
25	4.65 ± 0.34	0.85 ± 0.48	16.50 ± 1.25	3.02 ± 1.69	0.1040 ± 0.0025
125	4.71 ± 0.25	0.54 ± 0.31	16.35 ± 0.88	1.86 ± 1.07	0.1452 ± 0.0026

a Data from fitting to the ternary complex model (eq 1).

**Figure 1:**
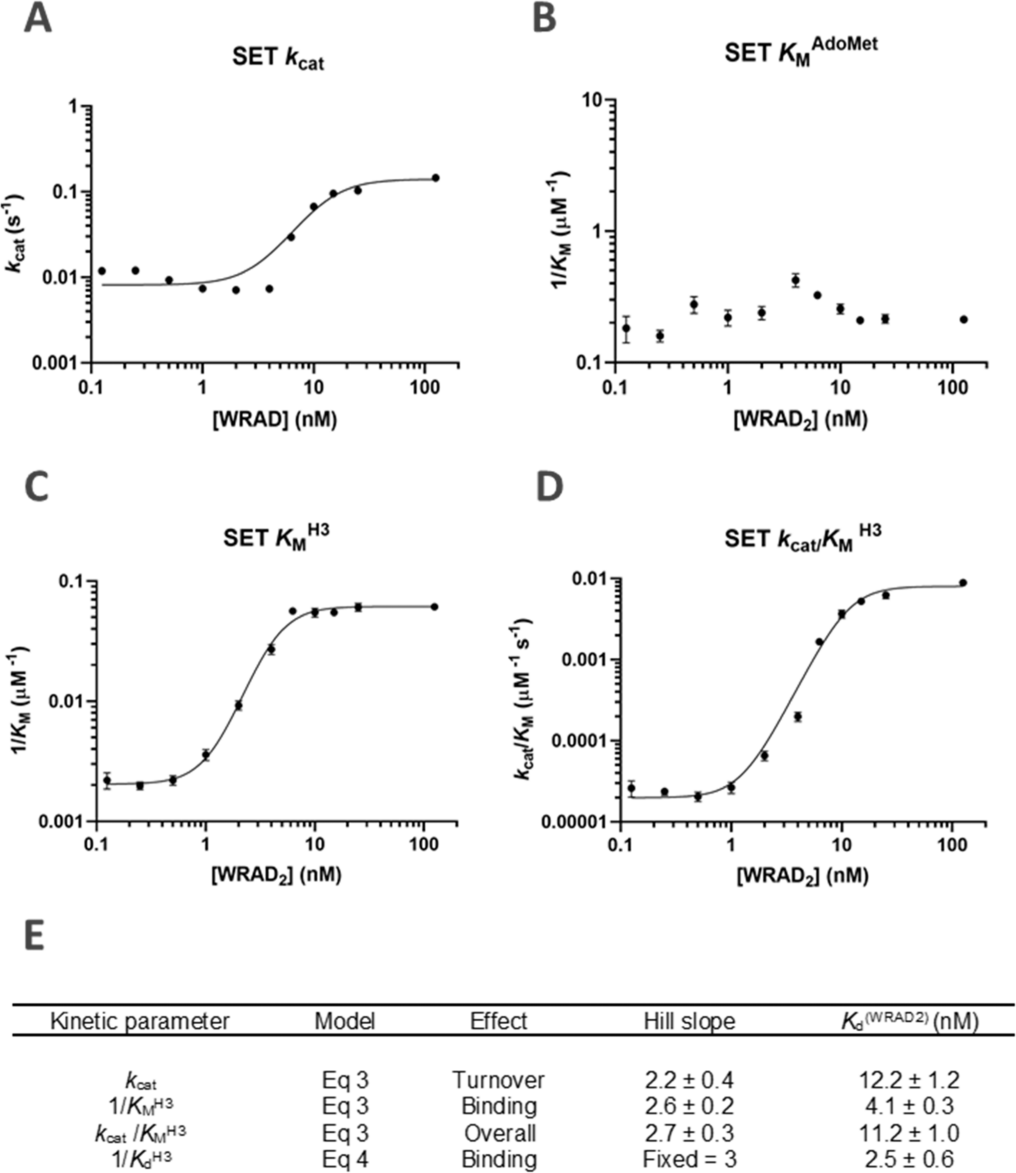
Fitting of the SET domain kinetics parameters measured as a function of WRAD2 concentration. (A) *k*_cat_ fitted to eq 3 gives a slope of 2.2 and a *K*_d_ of 12 nM. (B) AdoMet *K*_M_ is agnostic to WRAD2 concentration. (C) 1/*K*_M_ of H3_1–21_ fitted to eq 3 gives a slope of 2.6 and a *K*_d_ of 4.1 nM. (D) *k*_cat_/*K*_M_ of H3_1–21_ fitted to eq 3 gives a slope of 2.7 and a *K*_d_ 11.2 nM. (E) A table of measured Hill slopes and *K*_d_ values fitted to eqs 3 and 4.

A 30-fold increase in H3_1–21_ affinity and a 10-fold increase in *k*_cat_ were observed with increasing WRAD2 concentration. No significant change in AdoMet *K*_M_ was observed throughout the range of the titration, showing that WRAD2 has no effect on AdoMet binding when forming the ternary complex. Interestingly, the calculated *K*_d_ values for both AdoMet and H3_1–21_, which represent substrate binding to the free enzyme, decreased with increasing WRAD2 concentration. For both substrates, *K*_M_ and *K*_d_ had equivalent values in the absence of WRAD2, but the calculated *K*_d_ values reduced 10-fold at the highest WRAD2 concentration of 125 nM. Plotting the catalytic parameters as a function of WRAD2 concentration can give insights into the affinity and stoichiometry of any interactions with the SET domain and how WRAD2 affects the catalytic rate, substrate binding, and catalytic efficiency (Figure 1).

Data were fitted to a modified Hill model (eq 3), which is a cooperative model that reports a single *K*_d_ value, where multiple interactions can have similar affinities. The model also contains constant C, which allows for the basal activity of the SET domain in the absence of the WRAD2 complex. A plot of *k*_cat_ versus WRAD2 concentration showed a sigmoidal curve with a *K*_d_ = 12.2 ± 1.2 nM with a gradient of 2.2 ± 0.4. This WRAD2 dependence can only be explained by the involvement of at least two protein interactions. H3_1–21_ affinity plotted as 1/*K*_M_ showed a sigmoidal curve with a *K*_d_ = 4.1 ± 0.3 nM with a Hill slope = 2.6 ± 0.2. In addition, the overall rate constant, *k*_cat_/*K*_M_, was also plotted and again presented a sigmoidal curve with a *K*_d_ = 11.2 ± 1.0 nM with a Hill slope = 2.7 ± 0.3. Hill slopes of 2.6 and 2.7 for 1/*K*_M_ and *k*_cat_/*K*_M_, respectively, indicate the participation of two to three protein interactions. Attempts to fit the *k*_cat_, 1/*K*_M_, and *k*_cat_/*K*_M_ data sets to various models of independent or combinations of independent and cooperative binding yielded poor fits (data not shown). This is most likely due to the dissociation constants being too close in magnitude for the models to distinguish. AdoMet *K*_M_ showed no change with increasing WRAD2 concentration, so provided no information on the WRAD2 interaction. In addition, 1/*K*_d_ for H3_1–21_ was fitted to a cooperative three-binding-site model, where the Hill slope was set to a value of 3 (eq 4), giving a *K*_d_ of 2.5 ± 0.6 nM (Figure S4). This *K*_d_ is not significantly different from the *K*_d_ of 4.1 nM identified from the 1/*K*_M_ fit so may be the result of the same protein interactions. Again, attempts to fit these data to the modified Hill equation (eq 3) or various models of multiple binding sites were unsuccessful, but the cooperative three-binding-site model is consistent with Hill slopes observed from the *k*_cat_, 1/*K*_M_, and *k*_cat_/*K*_M_ fits. It should be noted that all of the measured interactions are well below the theoretical tight-binding limit of the assay, indicating that the active fraction of the SET domain must be below 4% of the total enzyme concentration. Individual substrate matrix global fits can be seen in Figure S5.

### Effect of Individual WRAD2 Components on SET Activity

**Figure 2:**
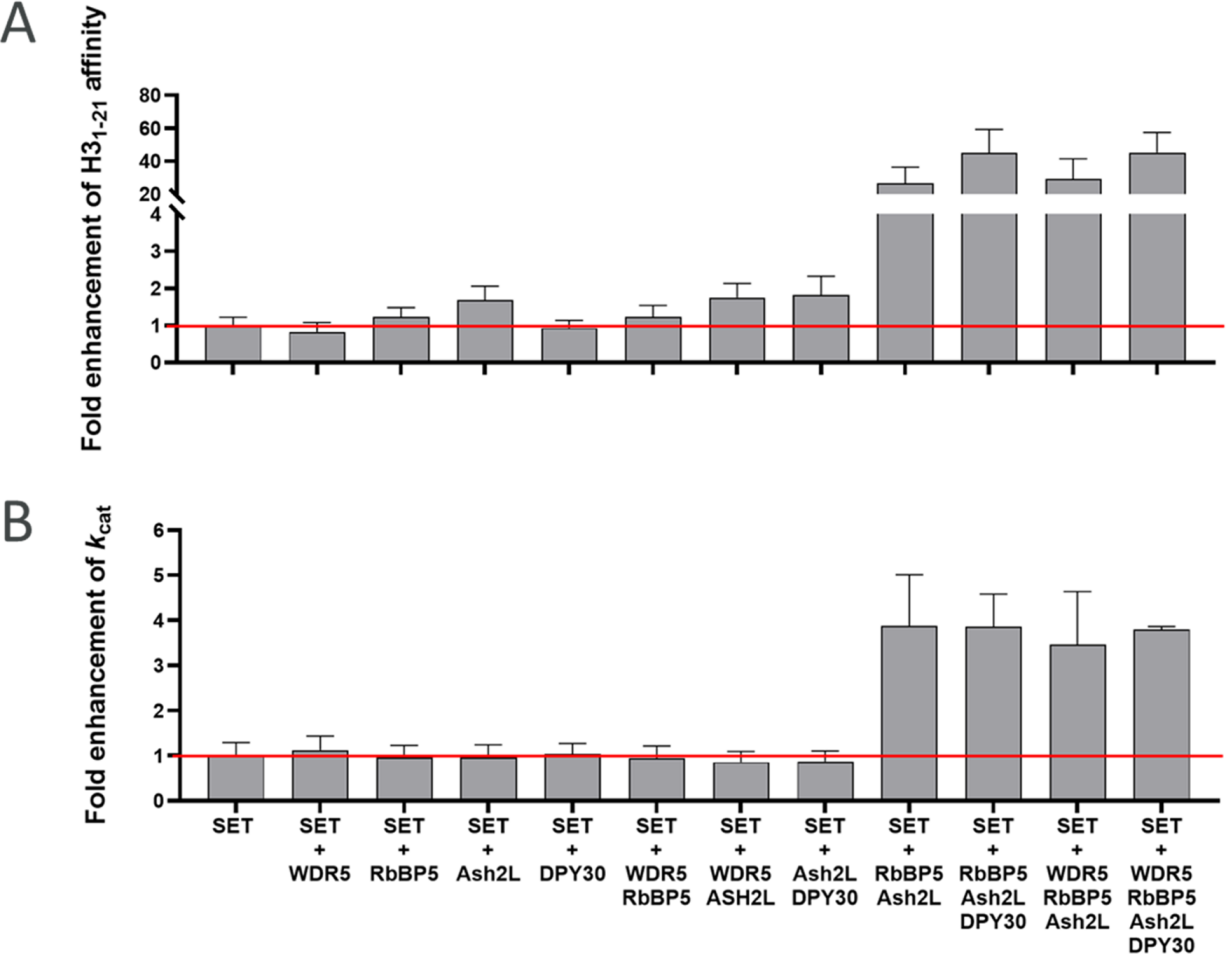
Bar chart showing the fold effects of individual and combinations of the WDR5, RbBP5, Ash2L, and DPY30 proteins on *k*_cat_ and *K*_M_ values of the SET domain. The red line indicates the basal level of the SET domain in isolation. (A) Ash2L has a 2-fold effect on H3_1–21_ affinity, but combinations of Ash2L and RbBP5 restore H3_1–21_*K*_M_ to the full SET/WRAD2 complex. (B) Individual WRAD2 components have no effect on SET domain *k*_cat_, but Ash2L and RbBP5 together form the core of the WRAD2 enhancement.

WDR5, RbBP5, Ash2L, and DPY30 were tested individually and in combination to identify the key WRAD2 components that interact with the SET domain (Figure 2).

As the *K*_M_ for AdoMet appeared to be agnostic to WRAD2 concentration, experiments were performed at a fixed AdoMet concentration of 5 μM while varying the H3_1–21_ concentration. Only Ash2L was identified to enhance peptide affinity 2-fold in isolation, whose effect was amplified 30-fold in the presence of RbBP5. No WRAD2 component in isolation had any stimulatory effect on *k*_cat_, but RbBP5 in combination with Ash2L enhanced *k*_cat_ to a level equivalent to the complete WRAD2 complex. These data showed that Ash2L and RbBP5 form two key interactions with the SET domain that affect both H3_1–21_ affinity and stimulation of *k*_cat_.

### SET/Ash2L/RbBP5 Minimal Complex

To investigate the minimal requirement of the Ash2L and RbBP5 interactions with the SET domain for efficient activity, a substrate matrix experiment was performed with the SET/Ash2L/RbBP5 complex using a truncated Ash2L construct (380-496-ISGRGS-539-598) and a 34 mer RbBP5 (330–363) peptide and fitted to eq 1. *k*_cat_ was ∼2-fold reduced and AdoMet *K*_M_ was equivalent to that measured with the full WRAD2 complex, but H3_1–21_*K*_M_ was 97.50 ± 3.1 μM cf. 16.5 ± 1.25 μM (Table 2).

**Table 2:** Steady-State Studies and Substrate Specificity of the hKMT2D SET Domain

complex	model	substrate	*K*_M_^(AdoMet)^ (μM)	*K*_d_^(AdoMet)^ (μM)	*K*_M_^(sub)^ (μM)	*K*_d_^(sub)^ (μM)	*k*_cat_ (s^–1^)	*k*_cat_/*K*_M_ (M^–1^ s^–1^)^c^
WRAD2^a^	eq 2	H3_1–21_	5.10 ± 0.32		18.12 ± 1.15		0.106 ± 0.003	5850 ± 537
WRAD2*	eq 1	H3_1–21_	4.65 ± 0.34	0.85 ± 0.48	16.50 ± 1.25	3.02 ± 1.69	0.104 ± 0.003	6364 ± 664
Ash2L/RbBP5^b^	eq 1	H3_1–21_	4.76 ± 0.20	7.63 ± 0.46	97.80 ± 3.10	156.70 ± 11.55	0.053 ± 0.001	542 ± 27
WRAD2	eq 1	Me1H3_1–21_	5.22 ± 0.32	3.76 ± 0.49	119.50 ± 5.70	85.40 ± 12.20	0.0054 ± 0.0001	45 ± 3
WRAD2	eq 1	Me2H3_1–21_	3.48 ± 0.36	0.04 ± 0.30	48.70 ± 4.38	5.91 ± 5.90	0.00038 ± 0.00001	8 ± 1
WRAD2	eq 5	Nuc^d^			0.99 ± 0.09		0.020 ± 0.002	20202 ± 3857
WRAD2	eq 5	Me1Nuc^d^			0.36 ± 0.08		0.00055 ± 0.00004	1528 ± 451
WRAD2	eq 5	Me2Nuc^d^			2.57 ± 1.1		0.00066 ± 0.00017	257 ± 176
WRAD2	eq 5	HeLa Nuc			1.1 ± 0.4		0.013 ± 0.001	12000 ± 4000

a Data from the 25 nM WRAD2 substrate matrix experiment.

b Truncated Ash2L and RbBP5 peptides.

c Catalytic efficiency calculated using substrate *K*_M_.

d Recombinant mononucleosomes.

These data confirm that this minimal complex shows the importance of the Ash2L and RbBP5 interactions, even in their truncated forms. Substrate matrix fits can be seen in Figure S6.

### Mono- and Dimethylated Peptide Substrates

As the KMT2D SET domain can catalyze mono-, di-, and trimethylation of H3K4, Me1H3_1–21_ and Me2H3_1–21_ peptides were used in substrate matrix experiments to determine substrate specificity (Table 2 and Figure S7). Using Me1H3_1–21_ as a substrate showed a 19-fold decrease in *k*_cat_ from a value of 0.104 s^–1^ for H3_1–21_ to 0.0054 s^–1^. A further 14-fold decrease was measured with Me2H3_1–21_ to 0.00038 s^–1^. Substrate *K*_M_ values for Me1H3_1–21_ and Me2H3_1–21_ were 119.6 and 48.7 μM, respectively. The decrease in *k*_cat_ and increase in substrate *K*_M_ on peptide methylation equated to a 140- and 815-fold decrease in catalytic efficiency (*k*_cat_/*K*_M_) with each methylation compared to the nonmethylated substrate H3_1–21_. These data indicate that nonmethylated H3_1–21_ is the preferred substrate in vitro.

### Nucleosome Substrates

To investigate any changes in substrate specificity under a potentially more physiologically relevant setting, recombinant mononucleosomes were used. Using recombinant mononucleosomes has the advantage of being able to control methyl marks on any given histone at any given position. These mononucleosomes provided substrates with specific methyl marks on the H3.1K4 residue. Overall, all nucleosome substrates were more efficient substrates than the peptides tested (Table 2 and Figure S8). Nevertheless, mononucleosomes followed a similar trend as to that observed for peptide substrates, with the catalytic efficiency decreasing with each methylation reaction from 2.0 × 10^4^ to 1.5 × 10^3^ and 2.6 × 10^2^ M^–1^ s^–1^ for mono-, di-, and trimethylation, respectively. These data suggest that whether methylating peptides or nucleosome substrates, the monomethylation reaction is the most efficient. The kinetic analysis of HeLa oligonucleosomes as a substrate gave a catalytic efficiency of 1.2 × 10^4^ M^–1^ s^–1^. The catalytic efficiency of the oligonucleosomes was in good agreement with the recombinant mononucleosomes. The reduction of efficiency relative to the unmethylated recombinant mononucleosomes was expected, given the possibility of increased methylation of the HeLa-derived oligonucleosomes at the H3K4 position. Caution should be taken while reporting the absolute values for *k*_cat_ and *K*_M_ from the nucleosome experiments, as the assays were limited by the concentration of the starting stocks, meaning that full titration curves could not always be measured.

### MALDI-ToF MS Time Course

**Figure 3:**
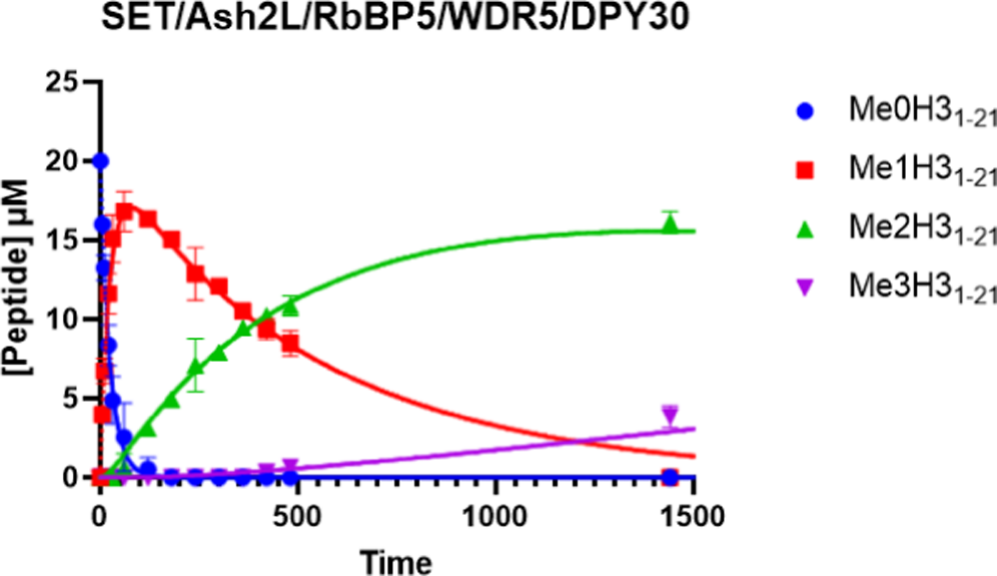
Distribution of substrates and products as a function of time for the SET/WRAD2 complex, consistent with a distributive mechanism. The assay used 200 nM 1:1 SET/WRAD2, 20 μM H3_1–21_, and excess 200 μM AdoMet. Fitting of the rates in KinTek Explorer v.10 showed a 20- and 10-fold decrease in the rate with each successive methylation.

To investigate the distributive or processive nature of the SET domain reaction, MALDI-ToF mass spectroscopy was used to monitor the peptide methylation state as a function of time (Figures 3 and S9 and Table 3).

**Table 3:** Methylation Rates Determined from MALDI-ToF Mass Spectrometry Time Courses Using KinTek Explorer v10. and Normalized to SET Domain Concentration

complex	[SET] nM	Me1H3_1–21_ (min^–1^)	Me2H3_1–21_ (min^–1^)	Me3H3_1–21_ (min^–1^)
SET	500	0.054 ± 0.011	0.002 ± ND	ND
SET/Ash2L	500	0.092 ± 0.023	0.002 ± 0.002	ND
SET/RbBP5	500	0.143 ± 0.022	0.0029 ± 0.0014	ND
SET/Ash2L/RbBP5	200	1.19 ± 0.62	0.051 ± 0.034	0.004 ± ND
SET/Ash2L/RbBP5/DPY30	200	1.26 ± 0.80	0.065 ± 0.031	0.006 ± 0.004
SET/Ash2L/RbBP5/WDR5	200	0.98 ± 0.74	0.046 ± 0.005	0.004 ± ND
SET/Ash2L/RbBP5/WDR5/DPY30	200	1.08 ± 0.64	0.055 ± 0.040	0.005 ± 0.006

**Scheme 1:**
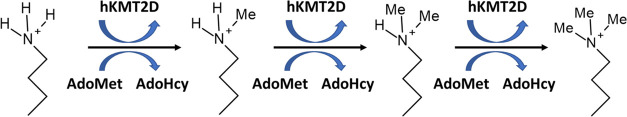
Depiction of Sequential Lysine Methylation Consistent with a Distributive Mechanism

In addition, these experiments can also provide insights into the effect of individual and combinations of WRAD2 proteins on product formation. Experiments used excess AdoMet at 200 μM, so the cofactor would not become limiting. Time courses in all conditions showed the consumption of the H3_1–21_ substrate and the formation of Me1H3_1–21_. Only after 24 h did the SET, SET/Ash2L, and SET/RbBP5 conditions show a significant quantity of Me2H3_1–21_ of ∼5 μM. The activity and product distribution significantly increased on the formation of the SET/Ash2L/RbBP5 complex, with the rapid consumption of H3_1–21_ within 60 min. After a significant concentration of Me1H3_1–21_ had accumulated, >75% of the total species, the evolution of Me2H3_1–21_ was observed with the accompanied consumption of Me1H3_1–21_. The same trend was observed for the formation of Me3H3_1–21_, requiring substantial accumulation of Me2H3_1–21_ before trimethylation would proceed. Fitting the progress curves in KinTek Explorer (Figure S9) showed that dimethylation was ∼20-fold slower than the monomethylation reaction and trimethylation was a further 10-fold less efficient, indicating that the SET domain is most efficient at monomethylation, consistent with steady-state experiments in Figure 2. Formation of higher complexes beyond SET/Ash2L/RbBP5 by the addition of WDR5 and DPY30 showed no measurable enhancement of activity or trimethylation. These data are also consistent with a distributive mechanism (Scheme 1), as a processive mechanism would show the consumption of H3_1–21_ and formation of Me3H3_1–21_ with little or no mono- or dimethylated product.

### Product Inhibitor Studies

**Figure 4:**
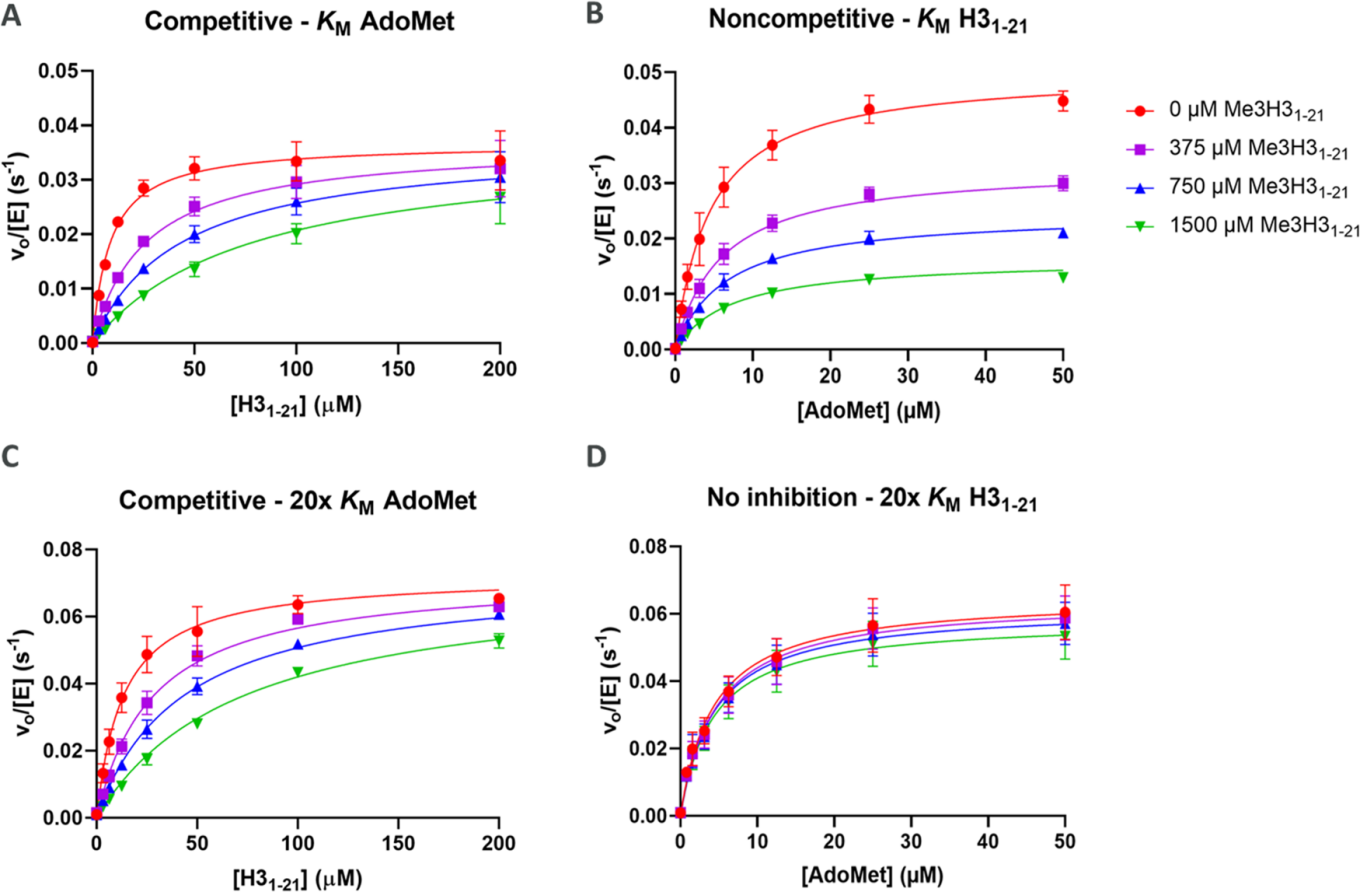
Representative product inhibitor data as a function of Me3H3_1–21_ concentration. (A, C) Me3H3_1–21_ is a competitive inhibitor when H3_1–21_ is varied at both *K*_M_ and 20× *K*_M_ AdoMet concentrations. (B) Me3H3_1–21_ is a noncompetitive inhibitor when AdoMet is varied at *K*_M_ concentration of H3_1–21_. (D) No inhibition by Me3H3_1–21_ when AdoMet is varied at 20× *K*_M_ H3_1–21_.

As the substrate matrix experiments often cannot confidently identify the enzyme mechanism, product inhibition studies were performed, using adenosyl-homocysteine (AdoHcy) and trimethylated H3 peptide (Me3H3_1–21_) as product inhibitors. Product inhibitors are part of the normal reaction coordinate and can bind to specific enzyme forms during the catalytic cycle. Me3H3_1–21_ was shown to be a competitive inhibitor when H3_1–21_ was the varied substrate and the concentration of AdoMet was fixed at both *K*_M_ and 20× *K*_M_. To check the validity of fitting to a competitive model, the data were fitted to the mixed inhibition model (eq 9) to determine the α value (α). When α is >1, the data tend toward competitive inhibition, when α is <1, the data tend toward uncompetitive inhibition, and when α = 1, the data show no bias toward either competitive or uncompetitive inhibition and are consistent with noncompetitive inhibition. When AdoMet was fixed at *K*_M_ and 20× *K*_M_, the α values for Me3H3_1–21_ were>1000 from both fits, confirming competitive inhibition. Me3H3_1–21_ showed noncompetitive inhibition when AdoMet was varied at fixed *K*_M_ H3_1–21_, but this inhibition was abolished when the fixed concentration of the H3_1–21_ peptide was increased to 20× *K*_M_ (Table 4 and Figures 4 and S10).

**Table 4:** Product and Dead-End Inhibitor Studies^a^

inhibitor	varied substrate	concentration of fixed substrate	inhibition pattern^a^	*K*_i_ (μM)	α^b^
AdoHcy	AdoMet	*K*_M_	C	4.95 ± 0.62	>1000
AdoHcy	AdoMet	20× *K*_M_	C	2.29 ± 0.20	6.80
AdoHcy	H3_1–21_	*K*_M_	NC	5.38 ± 0.41	0.56
AdoHcy	H3_1–21_	20× *K*_M_	no inhibition		
Me3H3_1–21_	AdoMet	*K*_M_	NC	608.0 ± 20.5	1.89
Me3H3_1–21_	AdoMet	20× *K*_M_	no inhibition		
Me3H3_1–21_	H3_1–21_	*K*_M_	C	168.8 ± 17.5	>1000
Me3H3_1–21_	H3_1–21_	20× *K*_M_	C	336.1 ± 21.8	>1000
sinefungin	AdoMet	*K*_M_	C	10.97 ± 0.46	21.10
sinefungin	H3_1–21_	*K*_M_	UC	10.33 ± 0.79	0.16
NleH3_1–21_	AdoMet	*K*_M_	UC	0.023 ± 0.001	0.09
NleH3_1–21_	H3_1–21_	*K*_M_	C	0.011 ± 0.007	862.10

a Parameters calculated from C = competitive, NC = noncompetitive, and UC = uncompetitive inhibition models using the Cleland nomenclature.

b α value determined from the mixed inhibition model.

AdoHcy inhibition was measured using concentrations up to a maximum concentration of 8 μM due to the limitations of the MTase-Glo technology. AdoHcy was a competitive inhibitor when varying AdoMet at both fixed *K*_M_ and 20× *K*_M_ H3_1–21_ concentrations, with the values of α being >1000 and 6.8, respectively. Noncompetitive inhibition was observed when H3_1–21_ was varied at *K*_M_ AdoMet. This inhibition was abolished when AdoMet was increased to 20× *K*_M_ (Figure S11). The competitive and noncompetitive product inhibition patterns observed are consistent with three enzyme mechanisms: Theorell–Chance, Ping–Pong, and rapid equilibrium random Bi–Bi with dead-end EAP and EBQ complexes.^41^

### Dead-End Inhibitor Studies

To further study the SET domain mechanism ascertained from the product inhibitor studies, dead-end inhibitors sinefungin and lysine 4 to the norleucine H3_1–21_ peptide (NleH3_1–21_) were used. In comparison to product inhibitors, dead-end inhibitors act as substrate analogues and divert the enzyme off the normal reaction coordinate. Hydrophobic mutations of H3K9, K27, and K36, by leucine, isoleucine, and methionine, have been reported to inhibit a number of KMTs and form the rational basis for using an inhibitory norleucine peptide.^42−44^ All experiments using dead-end inhibitors were performed with the nonvaried substrate concentration fixed at *K*_M_. Sinefungin and NleH3_1–21_ were fitted to a competitive model when AdoMet and H3_1–21_ were varied, respectively. The α values for sinefungin and NleH3_1–21_ were 21.1 and 862.2, respectively, confirming competitive inhibition. Unexpectedly, uncompetitive inhibition was observed for sinefungin and NleH3_1–21_ when H3_1–21_ and AdoMet were varied, respectively, and fitted to an uncompetitive model (Table 4 and Figures S12 and S13). Again, these data were fitted with a mixed inhibition model, which showed α values <1 of 0.16 and 0.09, confirming uncompetitive inhibition.

### SPR Binding Assays

To investigate the AdoMet and peptide binding properties of the SET domain, direct binding assays were performed using SPR. The minimal complex was used, as it gave better quality data due to its smaller size compared to the full SET/WRAD2 complex. AdoMet was found to bind the SET domain with an affinity of 9 ± 2 μM. This was in line with the *K*_M_ values measured during the steady-state experiments and gave confidence that the SET domain had not been adversely affected by immobilization. Peptide binding was measured both in the presence and absence of AdoMet using the dead-end inhibitor NleH3_1–21_ peptide. In the absence and presence of AdoMet, NleH3_1–21_ bound with affinities of 160 ± 57 and 10 ± 3 μM, respectively (Figure S14). These data indicate that NleH3_1–21_ binds with greater affinity in the presence of AdoMet in direct binding assays.

## Discussion

In this study, we aimed to address two main questions regarding human KMT2D (hKMT2D): First, what is the nature of the WRAD2 complex interaction with KMT2D? Second, what is the catalytic mechanism of the SET domain? Due to the size of hKMT2D (5537 residues), producing full-length proteins in sufficient quantities would have been technically demanding. With this in mind, we focussed on expressing amino acids 5308-5537 of the hKMT2D catalytic SET domain, including the WDR-interacting motif (Win motif), and the individual full-length WRAD2 components. Subsequently, intact mass spectrometry of the Win-SET domain revealed that the N-terminal Win motif was missing from the purified protein. The Win motif contains the conserved Arg5340 residue, which in multiple studies with KMT2A is proposed to form a central interaction with WDR5 and central to complex formation.^33^ Unsuccessful attempts were made to produce the intact Win-SET protein, including introducing point mutations around the cleavage site to inhibit proteolysis. Being unable to produce the intact Win-SET protein made performing a comparison study between SET and Win-SET domains impossible but would still provide information on the absolute requirement of the Win motif for WRAD2 modulation. Due to the absence of the Win motif, we refer to the catalytic subunit used here only as the SET domain.

To investigate the SET/WRAD2 interaction, SET domain kinetic parameters were measured at several WRAD2 concentrations, showing that the WRAD2 complex has a profound effect on the catalysis and binding of H3_1–21_. Fitting of *k*_cat_, 1/*K*_M_, and *k*_cat_/*K*_M_ as a function of WRAD2 concentration to the Hill equation returned gradients ranging from 2.2 to 2.7. This suggests that there are at least two interactions that elicit the enhanced response in H3_1–21_ affinity and catalytic activity, with measured affinities of ∼4 and 12 nM. The modulation of catalytic parameters is not the result of a single component of the WRAD2 complex, but the synergistic effect of multiple interactions, as illustrated by the Hill slopes >2. A single interaction modulating catalysis or substrate binding would have resulted in a Hill slope of near 1. Assays performed with the SET domain with individual and combinations of WDR5, RbBP5, Ash2L, and DPY30 identified Ash2L and RbBP5 as the two key proteins that together restore the SET domain function to that of the full WRAD2 complex. As Ash2L was observed to increase H3_1–21_ affinity in isolation and H3_1–21_ affinity responds to WRAD2 concentrations from 0.5 nM and above, we therefore assigned Ash2L a *K*_d_ of 4 nM. Using a similar process, we can assign RbBP5 a *K*_d_ of 12 nM, as the stimulation of *k*_cat_ does not occur until WRAD2 reaches a concentration of ∼2 nM and above. This highly active trimeric complex of SET/Ash2L/RbBP5 is consistent with observations from other studies.^45−48^ The relevance of this finding was also demonstrated in a substrate matrix experiment using the SET/Ash2L/RbBP5 minimal complex, consisting of a truncated Ash2L peptide (residues 539-496-ISGRGS-539-598) and an RbBP5 peptide (residues 330–363), based on the KMT2C study by Li et al.^45^*k*_cat_ was only 2-fold lower and the *K*_M_ value was 6-fold larger than those of the full SET/WRAD2 complex. The Win motif/WDR5 interaction is proposed to be the hub of complex formation in KMT2 proteins, mainly from studies conducted with KMT2A; but data presented here for the hKMT2D SET domain show that complexes can be formed in the absence of the Win motif. Cryo-electron microscopy has shown that the WRAD2 complex is dynamic in nature so can conceivably dissociate in solution at low concentrations and not titrate as a single entity.^35^ This structural information also formed the basis of the assumption that the SET domain associates with equimolar amounts of each of the WRAD2 components in solution. As all of the measured WRAD2 interactions are well below the tight-binding limit of the assay, this shows that the fraction of active enzyme is below 4%; therefore, the reported *k*_cat_ values in this study will be greatly underestimated. Without a tight-binding ligand, we cannot accurately measure the active fraction of enzyme in solution, although our values are in line with those previously reported by Zhang et al.^32^

A processive or distributive mechanism of the hKMT2D SET domain was investigated using MALDI-ToF mass spectrometry by monitoring the peptide substrate and product distributions as a function of time. This revealed a distributive mechanism, where the H3 peptide is monomethylated and released into solution before rebinding to carry out the second methylation reaction. This process is repeated to generate the trimethylated species. The release and rebinding of the methylated product must allow the reorientation of the lysine side chain to facilitate the second and third methylations (Scheme 1). The distributive mechanism is consistent with the kinetic models used in this study and also indicates that during initial rate experiments, the monomethylated peptide is the predominant form in solution. MALDI-ToF MS time course data and substrate matrix experiments using H3_1–21_, Me1H3_1–21_, and Me2H3_1–21_ peptides showed that the rate of each methylation reaction decreased ∼20- and 10-fold for each methylation step, respectively. We would postulate that a WRAD2 titration with Me1 and Me2H3_1–21_ substrates would show similar trends in the measured *k*_cat_ and *K*_M_ values as those with the H3_1–21_ substrate. This hypothesis is supported by the observation that all methylation reactions are stimulated by the formation of the SET/Ash2L/RbBP5 complex in the MALDI-ToF experiments. Nucleosomes proved to be the most efficient substrate in all methylation states compared to peptide substrates, driven much by the reduced substrate *K*_M_s, but mononucleosomes also followed a similar decline in catalytic efficiency upon methylation. This makes the hKMT2D SET domain an efficient monomethylase in in vitro. HeLa oligonucleosomes had a similar catalytic efficiency to the unmethylated recombinant mononucleosomes, indicating that the samples used were predominantly free of methylation at the H3 lysine 4 position. MALDI-ToF MS also reinforced the significance of the SET/Ash2L/RbBP5 complex, as WDR5 and DPY30 do not further enhance enzyme activity or methylation efficiency. It is unclear whether this is due to the absent Win motif denying WDR5 and DPY30 critical interactions but is consistent with the observations by Li et al.^45^ It is important to note that using the biochemical techniques described here can only identify interactions that alter the SET domain catalytic parameters but cannot report on potentially critical binding partners that act solely as scaffolds for protein–protein or protein–DNA interactions in vivo. A notable observation is how the hKMT2D SET domain shows remarkable similarity to wild-type EZH2, the catalytic KMT subunit of the PRC2 complex, in terms of the measured catalytic parameters from mono- to trimethylation, and its distributive mechanism.^49^

**Scheme 2:**
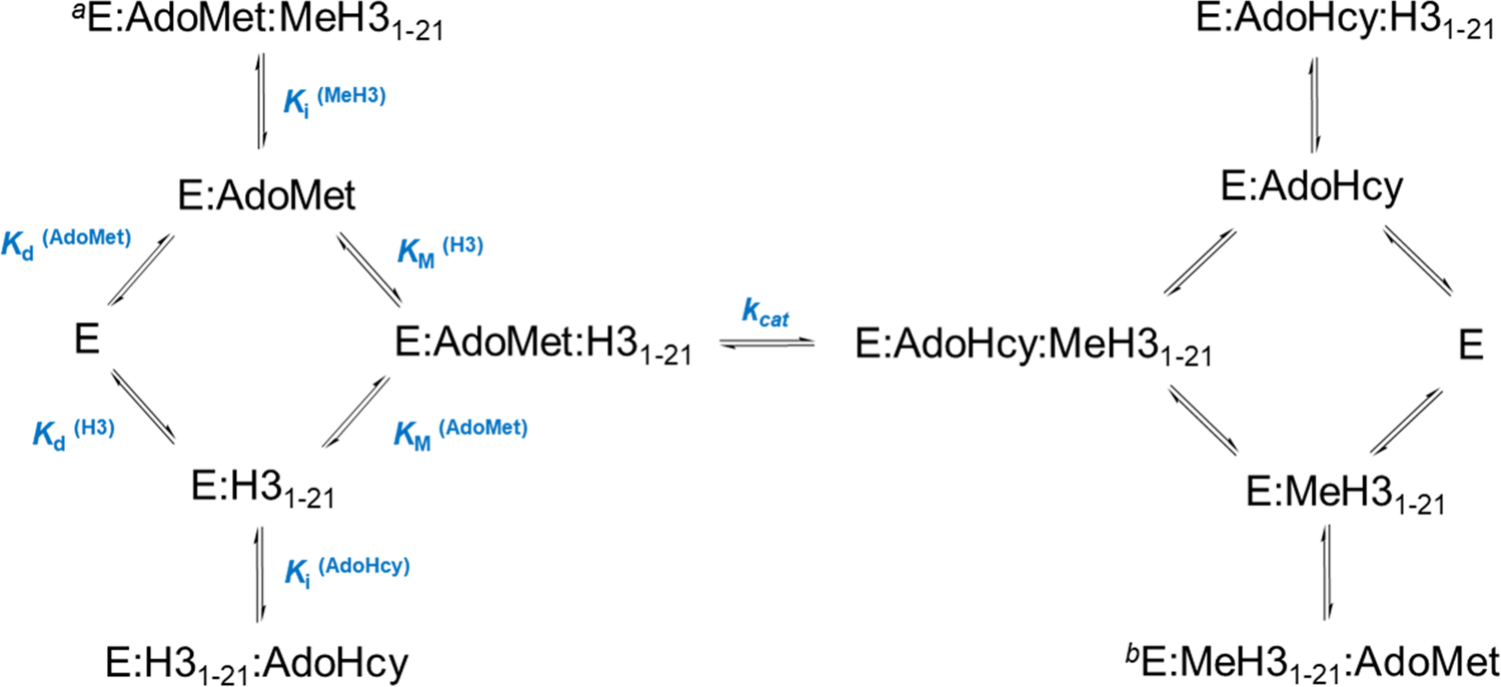
SET Domain Uses a Rapid Equilibrium Random Bi–Bi Mechanism with EAP and EBQ Dead-End Complexes, In proposed mechanisms, Me1H3_1–21_, Me2H3_1–21_, and Me3H3_1–21_ peptides can all act as product inhibitors but Me1 and Me2H3_1–21_ require binding in a specific orientation where the methyl group is directed toward the active site. Me1H3_1–21_ or Me2H3_1–21_ bound in an inhibitory conformation after catalysis and AdoHcy release. AdoMet binds before Me1 or Me2H3_1–21_ can be released to regenerate free enzymes. In blue are the parameters that can be determined from the steady state (*k*_cat_, *K*_d_ and *K*_M_) and product inhibitor experiments (*K*_i_).

Steady-state studies could not identify the enzyme mechanism solely from substrate matrix experiments. This is reflected here as the favored model changes, in a WRAD2 concentration-dependent manner, from ternary to a Ping–Pong model. This was the result of the calculated values for substrate *K*_d_ reducing with increasing WRAD2 concentration. When *K*_d_ becomes significantly small, then the *K*_d_*K*_M_^B^ term of the ternary complex equation (eq 1) tends to zero, and the equation collapses down to form the Ping–Pong model (eq 2). It is unlikely that an enzyme mechanism will change from the one that forms a ternary complex to the one that forms a covalent intermediate. Therefore, the ternary complex model satisfies all of the observed steady-state data. Moreover, a Ping–Pong mechanism would have suggested that the hKMT2D SET domain uses a novel mechanism among KMTs, with no published examples to date. To further probe the true enzyme mechanism, product and dead-end inhibitor studies were performed and the inhibition patterns were analyzed. The inhibition patterns can either be compared to published tables or be derived from first principles using Cleland’s rules to identify the enzyme mechanism.^41,50,51^ Published tables would indicate that the competitive and noncompetitive product inhibition patterns are consistent with the Theorell–Chance mechanism, Ping–Pong mechanism, and rapid equilibrium random Bi–Bi mechanism with dead-end EAP and EBQ complexes. Dead-end inhibitors produced distinctive competitive and uncompetitive patterns consistent with the Ping–Pong mechanism, which was surprising, as SET domain catalysis is widely accepted to occur through the nucleophilic attack of the AdoMet sulphonium center by the ε-amino group of lysine.^32^ For the Ping–Pong mechanism to hold, the product inhibitors Me3H3_1–21_ and AdoHcy cannot be competitive with their cognate substrates but would present as noncompetitive inhibition (Figure S15). This observation rules out Ping–Pong and Theorell–Chance as possible mechanisms. Although at first glance, the three possible mechanisms share the same product inhibition patterns, incorrect assignment of the product P and Q notation can have a profound effect on identifying the correct mechanism.^41,50^ In this instance, product inhibitors were sufficient to determine the SET domain mechanism. Uncompetitive inhibition has previously been observed with dead-end inhibitors with other SET domains, stating the formation of the E:AdoMet complex is a prerequisite for norleucine mimetics while not being required for lysine substrate binding.^42,52,53^ With this in mind, we suggest that the hKMT2D SET domain uses a rapid equilibrium random Bi–Bi mechanism with dead-end EAP and EBQ complexes (Scheme 2).

Furthermore, dead-end EAP and EBQ complexes are consistent with products Me3H3_1–21_ and AdoHcy competing with their cognate substrates. The potential dead-end complexes formed by the SET domain in Scheme 2 are made more complex by the fact that there are potentially three methylation events and therefore three products. The EAP and EBQ dead-end complexes in this case refer to E:H3_1–21_: AdoHcy and E:AdoMet:MeH3_1–21_, respectively, where MeH3_1–21_ can be the mono-, di-, or trimethylated peptide. An inhibitory EBQ complex arising from Me1H3_1–21_ or Me2H3_1–21_ would require binding in a specific orientation with the methyl group directed toward the catalytic site, otherwise a further methylation reaction will occur. We therefore propose that this inhibitory conformation is already satisfied when Me1H3_1–21_ or Me2H3_1–21_ remains bound to the enzyme after the methylation reaction and AdoHcy release. Therefore, if AdoMet binds before Me1H3_1–21_ or Me2H3_1–21_ is released, the E:AdoMet:Me1H3_1–21_ or E:AdoMet:Me2H3_1–21_ dead-end complex is formed. This is also consistent with the distributive mechanism reported here and a mechanism supported by Wang et al for PRMT5.^54^ A paper published by Zheng et al. proposes that the hKMT2D minimal complex uses a sequential Bi–Bi mechanism, where AdoMet is required to bind first.^37^ We believe that this discrepancy could in part be explained by the use of a slow substrate rather than a true product inhibitor. Zheng et al. used Me1H3_1–20_ as a product inhibitor, but we show that both Me1H3_1–21_ and Me2H3_1–21_ are substrates for SET/WRAD2. If the minimal complex can use Me1H3_1–20_ as a substrate, then the data, depending on the catalytic efficiency, can be skewed toward weak non- or uncompetitive inhibition. Indeed we have collected MALDI-ToF data with the minimal complex showing the evolution of Me2 and Me3H3_1–21_ products (data not shown). Performing the product inhibition experiments at both *K*_M_ and saturating fixed substrate concentrations would have been useful to resolve any ambiguity, as saturating H3_1–20_ would abolish Me1H3_1–20_ inhibition in a random mechanism. SPR data collected by ourselves showed that the NleH3_1–21_ peptide does indeed bind to the SET minimal complex, but with greater affinity in the presence of AdoMet, thus not only ruling out a random mechanism but also showing a disconnect between steady-state and direct binding assays using dead-end inhibitors. Conversely, we cannot rule out that the hKMT2D minimal complex uses a different mechanism to the full SET/WRAD2 complex.

In summary, there are two critical WRAD2 components, Ash2L and RbBP5, both with low nanomolar affinities for the hKMT2D SET domain that modulate catalytic activity and substrate affinity. The Win motif is not crucial for SET/WRAD2 complex formation. Finally, the hKMT2D SET domain uses a rapid equilibrium Bi–Bi mechanism with EAP and EBQ dead-end complexes. It is hoped that this greater mechanistic insight into hKMT2D can help guide drug discovery strategies. The knowledge of the possible enzyme forms available during the catalytic cycle and the involvement of the key protein–protein interactions enable the rational design of assays to target defined enzyme complexes by small-molecule inhibitors.

## Supporting Information Available

Amino acid sequences of human KMT2D and WRAD2 complex components; WRAD2 titration experimental conditions; intact mass spec of the Win-SET protein; steady-state plots and fits to the ternary complex and Michaelis–Menten models; MALDI-ToF MS time courses; AdoHcy and Me3H3_1–21_ product inhibitor plots and fits; sinefungin and NleH3_1–21_ dead-end inhibitor plots and fits; and hypothetical hKMT2D Ping–Pong mechanism (PDF)

## Accession Codes

KMT2D, UniProt ID O14686; WDR5, UniProt ID P61964; RbBP5, UniProt ID Q15291; Ash2L, UniProt ID Q9UBL3; DPY30, UniProt ID Q9C005

## Abbreviations

hKMT2D: human lysine methyltransferase 2D
AdoMet: *S*-adenosyl methionine
AdoHcy: *S*-adenosyl homocysteine
MALDI-ToF MS: matrix-assisted laser desorption ionization time-of-flight mass spectrometry
MTase-Glo: methyltransferase-Glo
