# Supplementary material for: Dissecting the Kinetic Mechanism of Human Lysine Methyltransferase 2D and Its Interactions with the WRAD2 Complex
Source: Biochemistry. 2022 Sep 7;61(18):1974–87. doi: 10.1021/acs.biochem.2c00385 (PMC9494746; doi:10.1021/acs.biochem.2c00385)
Supplement: Supplementary file 1 — bi2c00385_si_001.pdf [file bi2c00385_si_001.pdf]

Supporting information for:

Dissecting the Kinetic Mechanism of Human Lysine Methyltransferase 2D and its Interactions with the WRAD2 Complex

Lucy V. Edwardes[1α], Sarah J. Caswell[1α], Mariacarmela Giurrandino[1], Xiang Zhai[2], Andrea Gohlke[3], Demetrios H. Kostomiris[2], Hannah K. Pollard[1], Alexander Pflug[3], Gregory R. Hamm[4], Kate V. Jervis[1], Paul N. Clarkson[1] and Karl Syson*[1]

[1] Discovery Biology, Discovery Sciences, BioPharmaceuticals, R&D, AstraZeneca, Cambridge, CB4 0WG, UK

[2] Mechanistic and Structural Biology, Discovery Sciences, BioPharmaceuticals, R&D, AstraZeneca, Boston, MA 02210, USA

[3] Mechanistic and Structural Biology, Discovery Sciences, BioPharmaceuticals, R&D, AstraZeneca, Cambridge, CB4 0WG, UK

[4] Imaging and Data Analytics, Clinical Pharmacology and Safety Sciences, BioPharmaceuticals

R&D, AstraZeneca, Cambridge, CB4 0WG, UK

[α] These authors contributed equally to this work

Correspondence should be addressed to: Dr. Karl Syson, AstraZeneca, Darwin Building, 310 Milton Road,

Cambridge, CB4 0WG, United Kingdom.

Email: karl.syson@astrazeneca.com

His-SET (5382-5537)

```
MGHHHHHHGG GENLYFQGS
      5391       5401       5411       5421       5431       5441
HSKSSQYRRL RTEWKNNVYL ARSRIQGLGL YAAKDLEKHT MVIEYIGTII RNEVANRREK
      5451       5461       5471       5481       5491       5501
IYEEQNRGIY MFRINNEHVI DATLTGGPAR YINHSCAPNC VAEVVTFDKE DKIIIISSRR
      5511       5521       5531
IPKGEELTYD YQFDFEDDQH KIPCHCGAWN CRKWMN
```

Win-SET (5308-5537)

```
MGHHHHHHGG GENLYFQGSHM
      5317       5327       5337       5347       5357       5367
LPGVESCQNY LFRYGRHPLM ELPLMINPTG CARSEPKILT HYKRPHTLNS TSMSKAYQST
      5377       5387       5397       5407       5417       5427
FTGETNTPYS KQFVHSKSSQ YRRLRTEWKN NVYLARSRIQ GLGLYAAKDL EKHTMVIEYI
      5437       5447       5457       5467       5477       5487
GTIIRNEVAN RREKIYEEQN RGIYMFRINN EHVIDATLTG GPARYINHSC APNCVAEVVT
      5497       5507       5417       5427       5437
FDKEDKIIII SSRRIPKGEE LTYDYQFDFE DDQHKIPCHC GAWNCRKWMN
```

WDR5 (1-334)

```
MGHHHHHHGG GENLYFQGSHM
        10         20         30         40         50         60
MATEEKKPET EAARAQPTPS SSATQSKPTP VKPNYALKFT LAGHTKAVSS VKFSPNGEWL
        70         80         90        100        110        120
ASSSADKLIK IWGAYDGKFE KTISGHKLGI SDVAWSSDSN LLVSASDDKT LKIWDVSSGK
       130        140        150        160        170        180
CLKTLKGHSN YVFCCNFNPQ SNLIVSGSFD ESVRIWDVKT GKCLKTLPAH SDPVSAVHFN
       190        200        210        220        230        240
RDGSLIVSSS YDGLCRIWDT ASGQCLKTLI DDDNPPVSFV KFSPNGKYIL AATLDNTLKL
       250        260        270        280        290        300
WDYSKGKCLK TYTGHKNEKY CIFANFSVTG GKWIVSGSED NLVYIWNLQT KEIVQKLQGH
       310        320        330
TDVVISTACH PTENIIASAA LENDKTIKLW KSDC
```

DPY30 (1-99)

```
MGHHHHHHGG GENLYFQGSHM
        10         20         30         40         50         60
MEPEQMLEGQ TQVAENPHSE YGLTDNVERI VENEKINAEK SSKQKVDLQS LPTRAYLDQT
        70         80         90
VVPILLQGLA VLAKERPPNP IEFLASYLLK NKAQFEDRN
```

Ash2L (1-534)

```
MGHHHHHHGG GENLYFQGS
        10         20         30         40         50         60
MDTQAGSVDE ENGRQLGEVE LQCGICTKWF TADTFGIDTS SCLPFMTNYS FHCNVCHHSG
        70         80         90        100        110        120
NTYFLRKQAN LKEMCLSALA NLTWQSRTQD EHPKTMFSKD KDIIPFIDKY WECMTTRQRP
       130        140        150        160        170        180
GKMTWPNNIV KTMSKERDVF LVKEHPDPGS KDPEEDYPKF GLLDQDLSNI GPAYDNQKQS
       190        200        210        220        230        240
SAVSTSGNLN GGIAAGSSGK GRGAKRKQQD GGTTGTTKKA RSDPLFSAQR LPPHGYPLEH
       250        260        270        280        290        300
PFNKDGYRYI LAEPDPHAPD PEKLELDCWA GKPIPGDLYR ACLYERVLLA LHDRAPQLKI
       310        320        330        340        350        360
SDDRLTVVGE KGYSMVRASH GVRKGAWYFE ITVDEMPPDT AARLGWSQPL GNLQAPLGYD
       370        380        390        400        410        420
KFSYSWRSKK GTKFHQSIGK HYSSGYGQGD VLGFYINLPE DTETAKSLPD TYKDKALIKF
       430        440        450        460        470        480
KSYLYFEEKD FVDKAEKSLK QTPHSEIIFY KNGVNQGVAY KDIFEGVYFP AISLYKSCTV
       490        500        510        520        530
SINFGPCFKY PPKDLTYRPM SDMGWGAVVE HTLADVLYHV ETEVDGRRSP PWEP
```

Ash2L truncate (380-496-ISGRGS-539-598)

```
MHHHHHHGGG ENLYFQGS
       389        399        409        419        429        439
RVLLALHDRA PQLKISDDRL TVVGEKGYSM VRASHGVRKG AWYFEITVDE MPPDTAARLG
       449        459        469        479        489
WSQPLGNLQA PLGYDKFSYS WRSKKGTKFH QSIGKHYSSG YGQGDVLGFY INLPEDTISG
       545        555        565        575        585        595
RGSSEIIFYK NGVNQGVAYK DIFEGVYFPA ISLYKSCTVS INFGPCFKYP PKDLTYRPMS

DMG
```

RbBP5 (1-538)

```
MHHHHHHGGG ENLYFQGSHM
        10         20         30         40         50         60
MNLELLESFG QNYPEEADGT LDCISMALTC TFNRWGTLLA VGCNDGRIVI WDFLTRGIAK
        70         80         90        100        110        120
IISAHIHPVC SLCWSRDGHK LVSASTDNIV SQWDVLSGDC DQRFRFPSPI LKVQYHPRDQ
       130        140        150        160        170        180
NKVLVCPMKS APVMLTLSDS KHVVLPVDDD SDLNVVASFD RRGEYIYTGN AKGKILVLKT
       190        200        210        220        230        240
DSQDLVASFR VTTGTSNTTA IKSIEFARKG SCFLINTADR IIRVYDGREI LTCGRDGEPE
       250        260        270        280        290        300
PMQKLQDLVN RTPWKKCCFS GDGEYIVAGS ARQHALYIWE KSIGNLVKIL HGTRGELLLD
       310        320        330        340        350        360
VAWHPVRPII ASISSGVVSI WAQNQVENWS AFAPDFKELD ENVEYEERES EFDIEDEDKS
       370        380        390        400        410        420
EPEQTGADAA EDEEVDVTSV DPIAAFCSSD EELEDSKALL YLPIAPEVED PEENPYGPPP
       430        440        450        460        470        480
DAVQTSLMDE GASSEKKRQS SADGSQPPKK KPKTTNIELQ GVPNDEVHPL LGVKGDGKSK
       490        500        510        520        530
KKQAGRPKGS KGKEKDSPFK PKLYKGDRGL PLEGSAKGKV QAELSQPLTA GGAISELL
```

Figure S1. Amino acid sequence of constructs used in this study with 6x His tag (Green) and TEV cleavage site (Red) and Ash2L linker in (Brown).

20793.7 Da

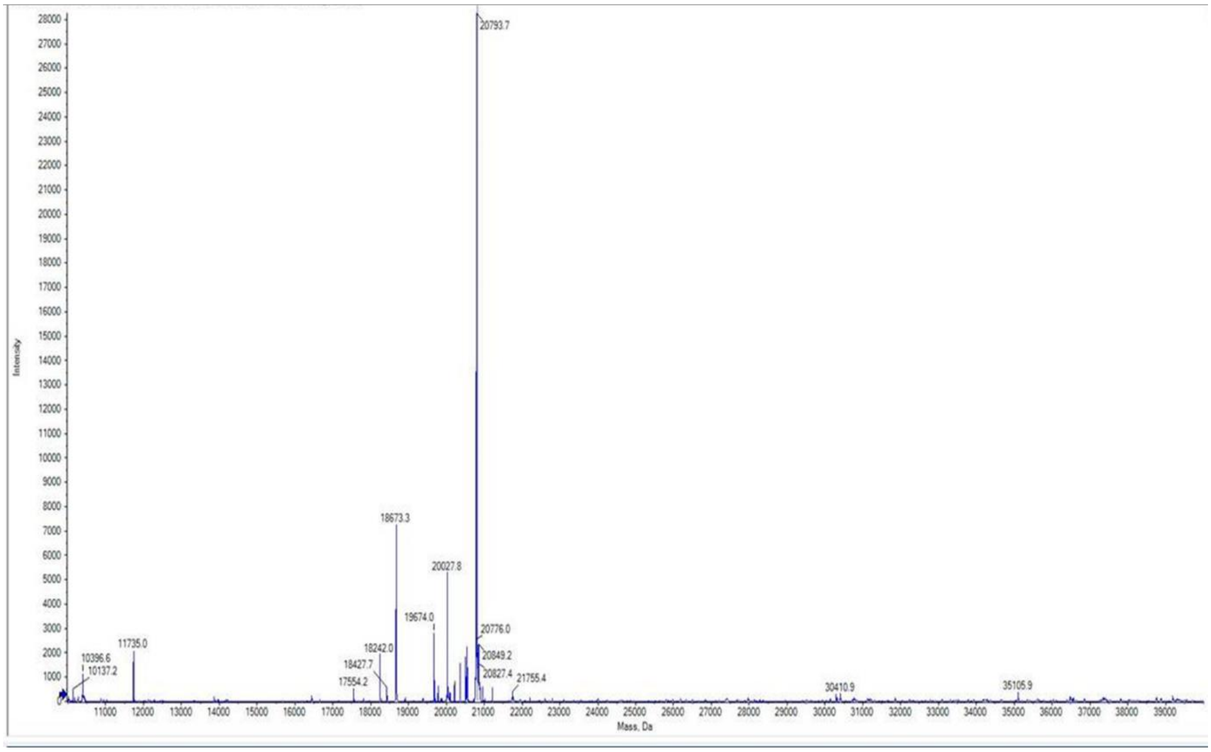

Figure S2. Intact mass spec of the Win-SET protein. Expected mass = 29,266 Da, observed mass = 20,794 Da. Observed mass corresponds to the loss of histidine and TEV tags and amino acids residues 5,308 to 5,360.

[WRAD2] (nM)	[SET] (nM)	[SAM] (μM)	$[H3_{1\text{-}21}]$ (μM)	Timepoints (min)
0	125	0-50	0-1000	0-50
0.125	125	0-50	0-1000	0-50
0.25	125	0-50	0-1000	0-50
0.5	125	0-50	0-1000	0-50
1	125	0-50	0-750	0-50
2	125	0-50	0-500	0-50
4	125	0-50	0-250	0-40
6.25	30	0-50	0-250	0-40
10	30	0-50	0-200	0-30
15	30	0-50	0-200	0-30
25	30	0-50	0-200	0-30
125	30	0-50	0-200	0-20

Figure S3. Steady state assay conditions used for the WRAD2 Complex Titration.

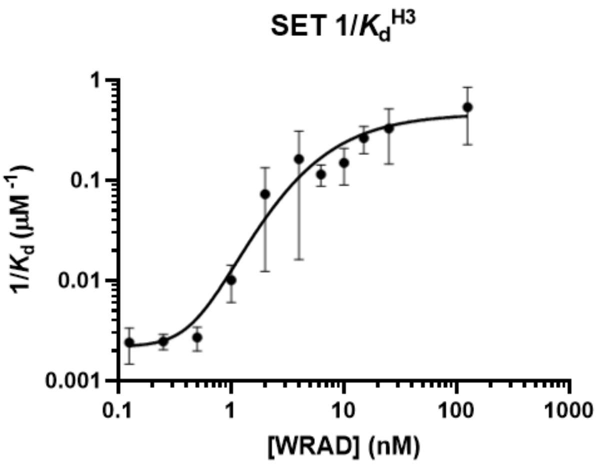

Figure S4. Plot of $1/K_d$ for $H3_{1-21}$ peptide as calculated from the ternary complex model. Experiments were performed in triplicate and expressed as the mean ± SD. Data was fit in GraphPad Prism v9.1 to a cooperative model were the Hill constant was fixed to a value of three.

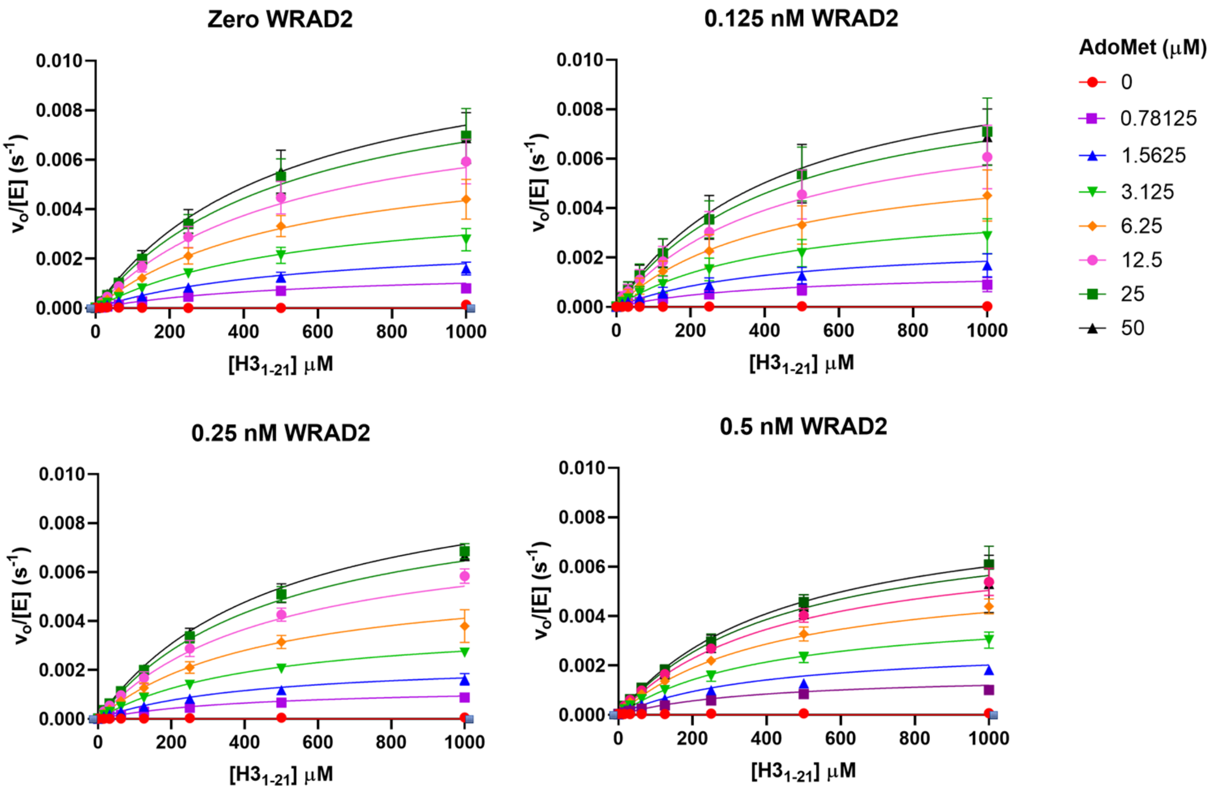

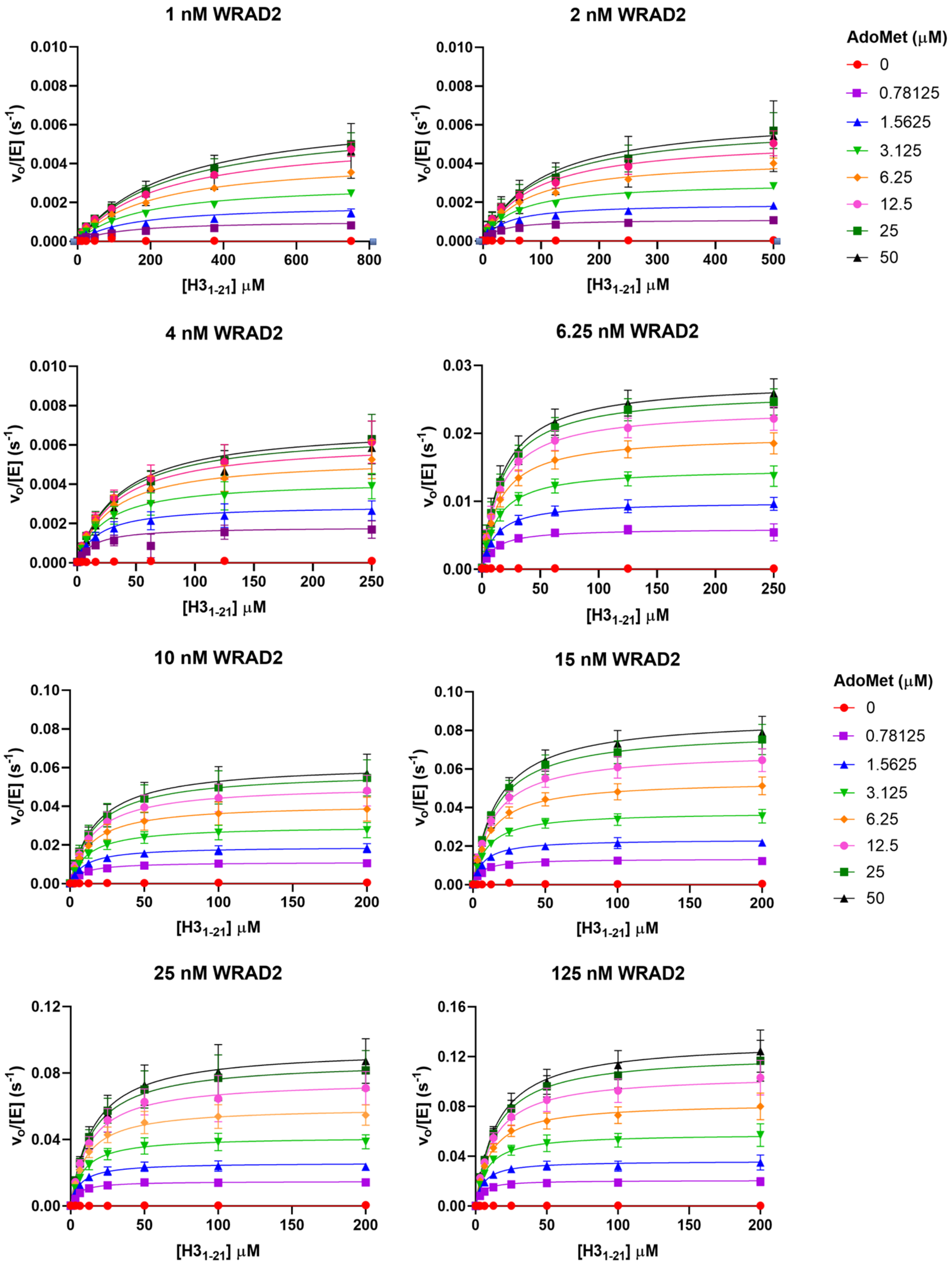

Figure S5. Substrate matrix experiments as a function of WRAD2 complex ranging from zero to 125 nM WRAD2 with SET domain. Experiments were performed in triplicate and expressed as the mean ± SD. Data was fit globally to the ternary complex model in GraphPad Prism v9.1.

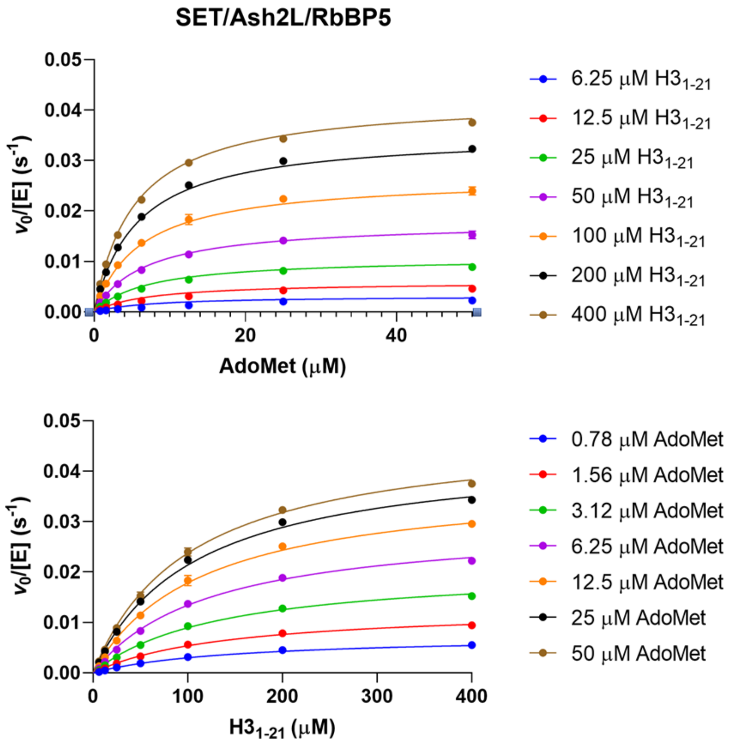

Figure S6. Substrate matrix experiment using the SET/Ash2L/RbBP5 minimal complex with $H3_{1-21}$ peptide. Experiments were performed in triplicate and expressed as the mean ± SD. Data was fit globally to the ternary complex model in GraphPad Prism v9.1.

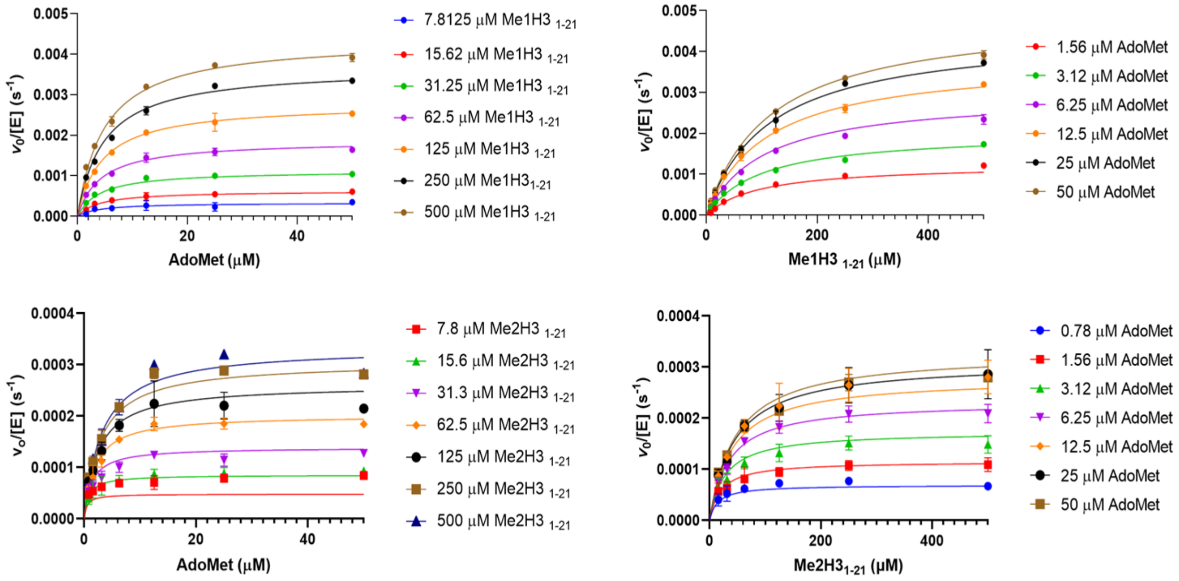

Figure S7. Substrate matrix experiment using SET/WRAD2 complex with $Me1H3_{1-21}$ and $Me2H3_{1-21}$ substrates. Experiments were performed in triplicate and expressed as the mean ± SD. Data was fit globally to the ternary complex model in GraphPad Prism v9.1.

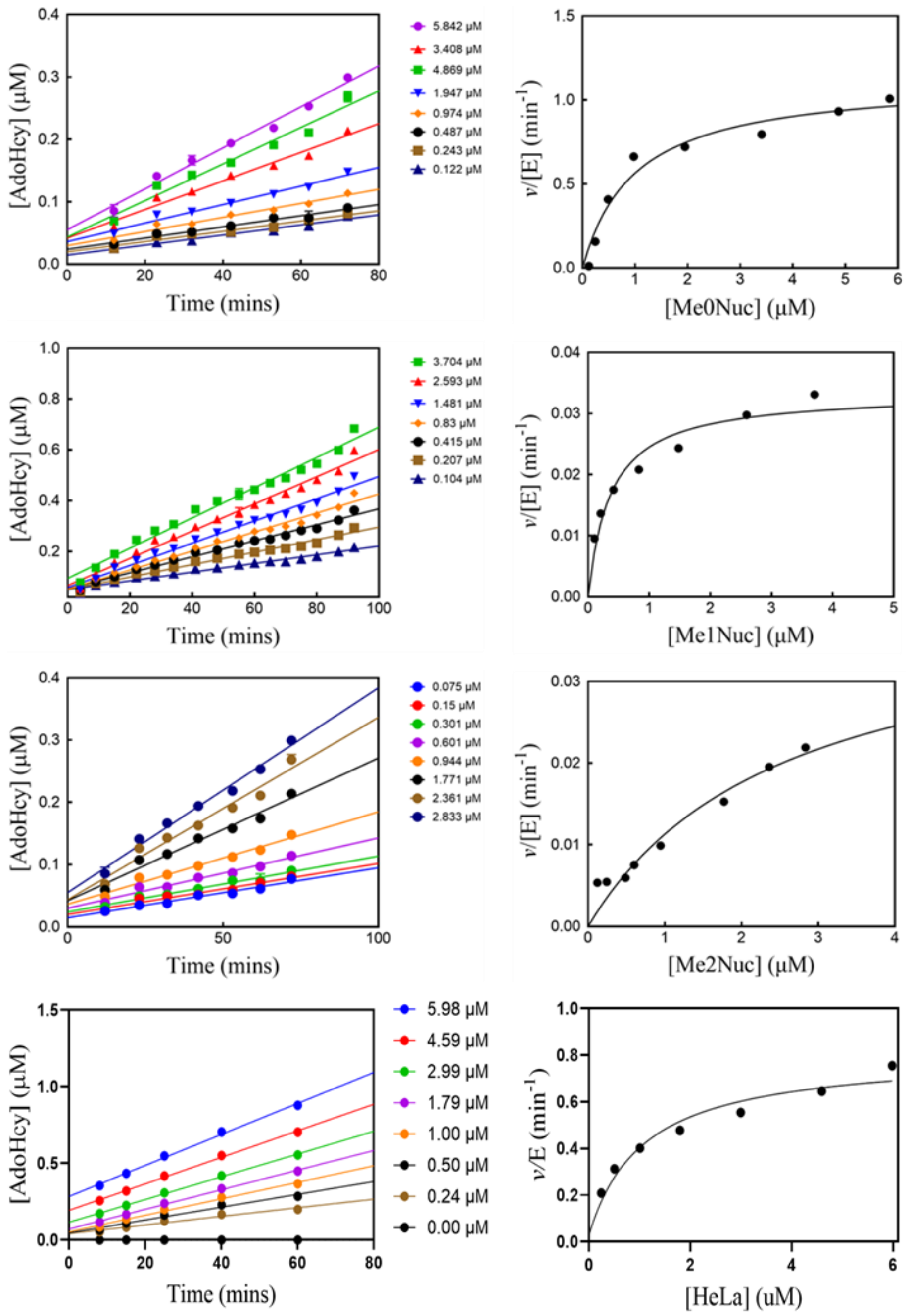

Figure S8. Determination of the k_{cat} and K_M values using SET/WRAD2 complex with Me0, Me1 and Me2 recombinant mononucleosomes and HeLa oligonucleosomes. Experiments were performed in triplicate and expressed as the mean ± SD. Data was fit to the Michaelis-Menten equation in GraphPad Prism v9.1.

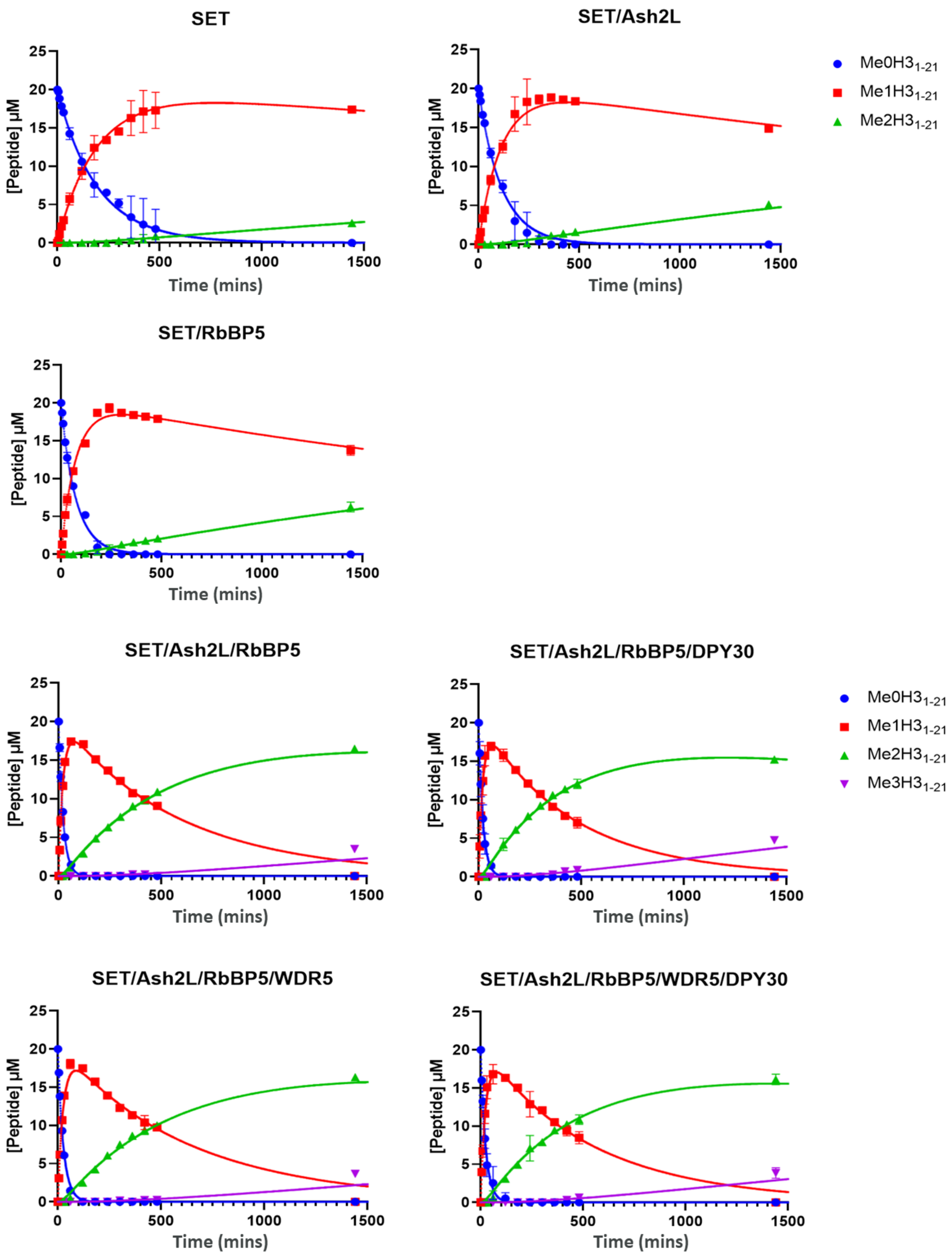

Figure S9. MALDI-ToF mass spectrometry showing product distribution as a function of time with the SET domain and combinations of SET and WRAD2 proteins. Experiments were performed in triplicate and expressed as the mean ± SD. Time courses were fit in KinTek Explorer v10 to a sequential methylation model.

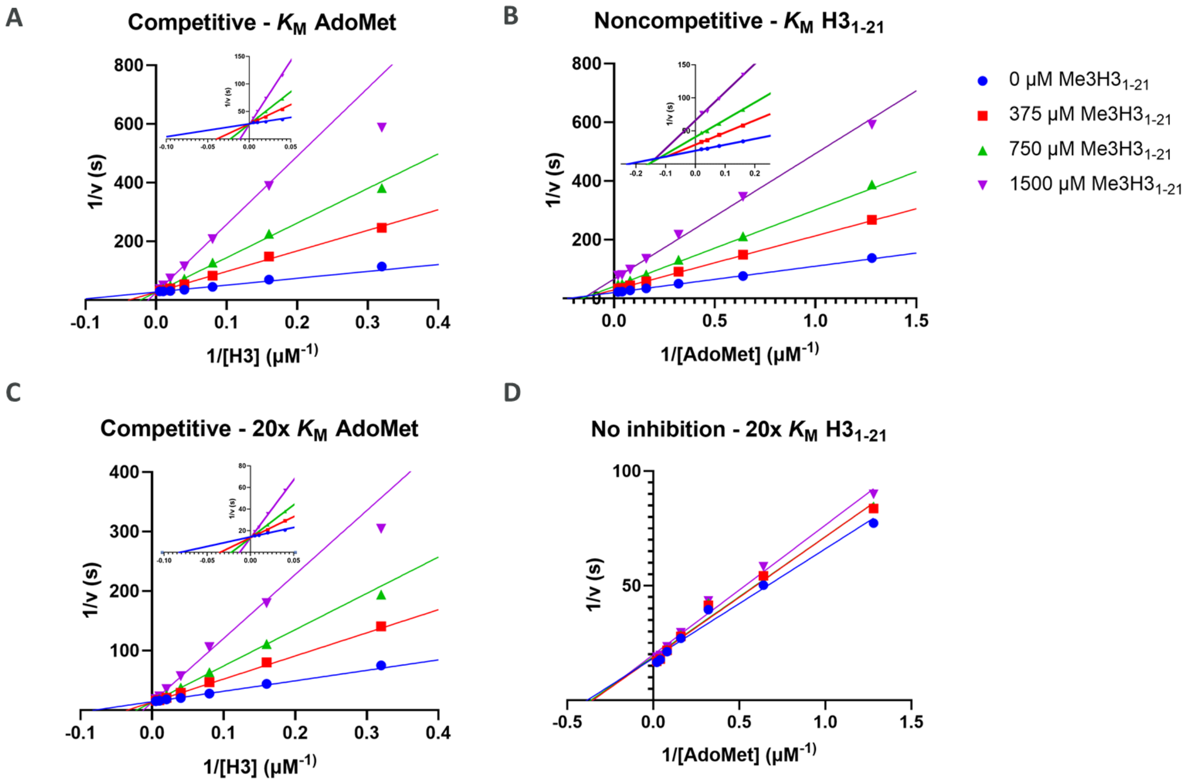

Figure S10. Double reciprocal plots of Me3H3$_{1-21}$ product inhibition matrix experiments with SET/WRAD2 complex at K_M and 20x K_M fixed substrate concentrations. Fits were generated by calculating $1/k_{cat}$ and K_M/k_{cat} after fitting individual curves to the Michealis-Menten equation with non-linear regression. (A) and (C) shows Me3H3$_{1-21}$ is a competitive inhibitor when H3$_{1-21}$ is varied at both K_M and 20x K_M AdoMet concentrations (B) shows no noncompetitive inhibition by Me3H3$_{1-21}$ when AdoMet is varied at K_M H3$_{1-21}$. (D) shows no inhibition of Me3H3$_{1-21}$ when AdoMet is varied at 20x K_M H3$_{1-21}$. Experiments were performed in triplicate. Data was analysed using GraphPad Prism v9.1.

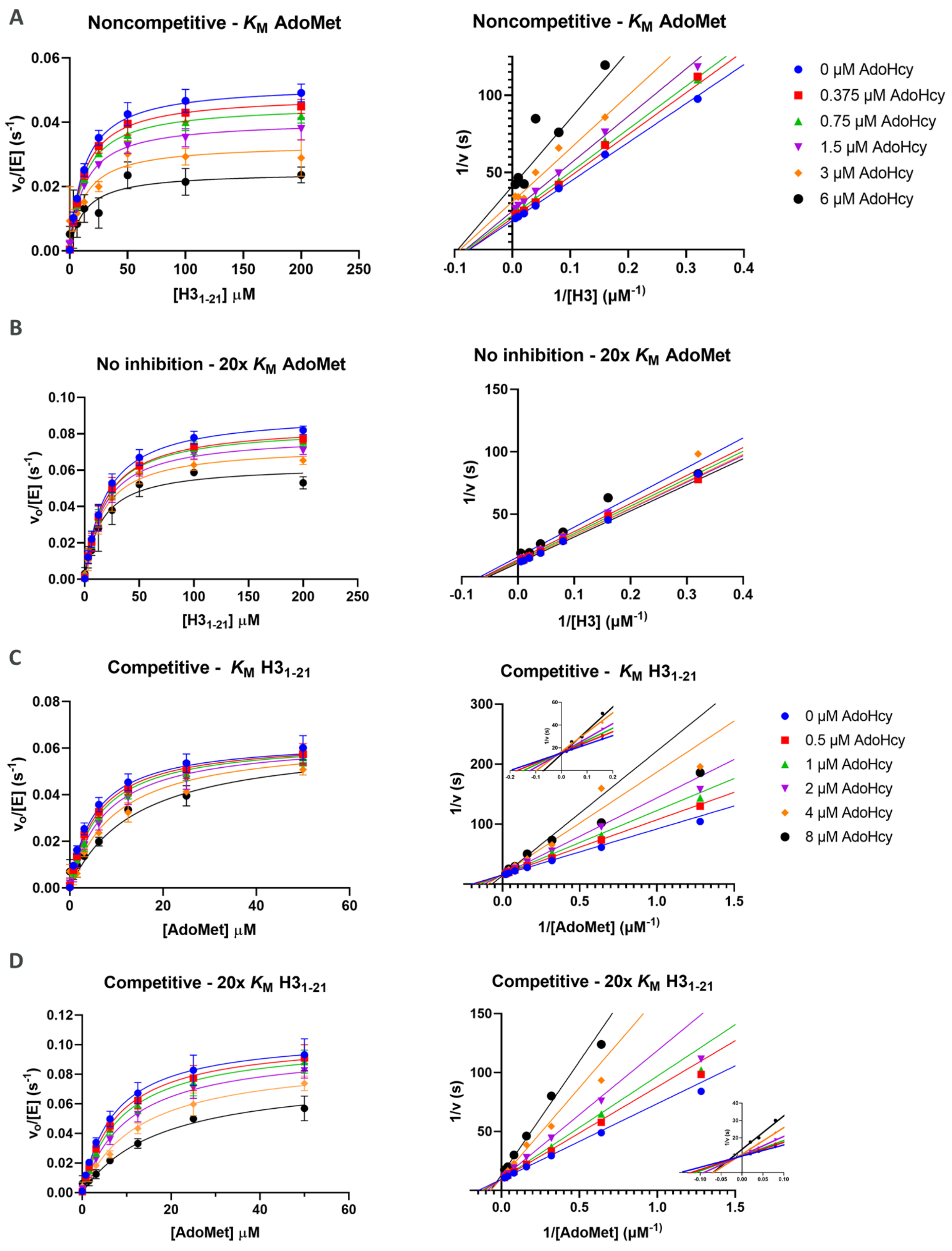

Figure S11. Non-linear and double reciprocal plots of AdoHcy product inhibition experiments with SET/WRAD2 at K_M and 20x K_M fixed substrate. Double reciprocal fits were generated by calculating $1/k_{cat}$ and K_M/k_{cat} after fitting individual curves to the Michealis-Menten equation with non-linear regression. (A) shows noncompetitive inhibition of AdoHcy when $H3_{1\text{-}21}$ is varied at K_M AdoMet. (B)

shows no inhibition by AdoHcy when H3$_{1-21}$ is varied at 20x K_M AdoMet. (C) and (D) shows AdoHcy is a competitive inhibitor when AdoMet is varied at both K_M and 20x K_M H3$_{1-21}$ concentrations Experiments were performed in triplicate and expressed as the mean ± SD. Data was analysed using GraphPad Prism v9.1.

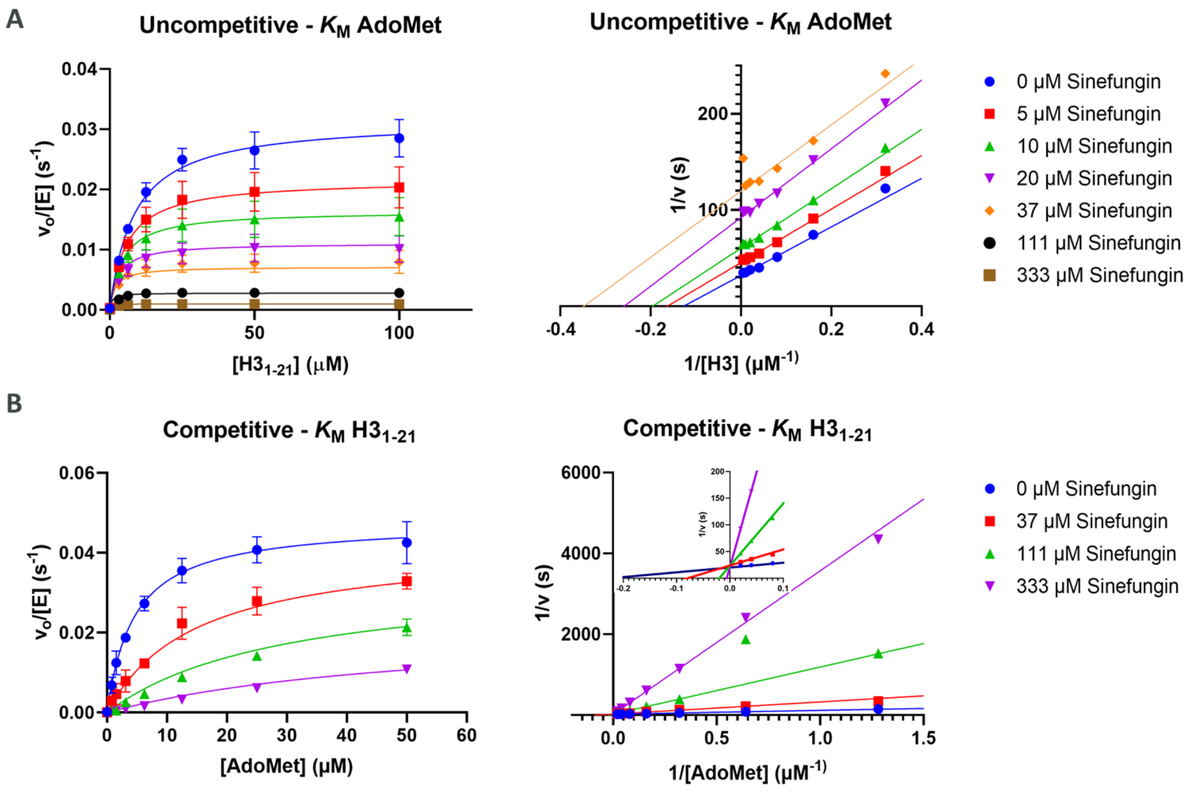

Figure S12. Non-linear and double reciprocal plots of sinefungin dead-end inhibitor experiments with SET/WRAD2 at K_M fixed substrate. Double reciprocal fits were generated by calculating $1/k_{cat}$ and K_M/k_{cat} after fitting individual curves to the Michealis-Menten equation with non-linear regression. (A) shows uncompetitive inhibition of sinefungin when H3$_{1-21}$ is varied at K_M AdoMet. (B) shows competitive inhibition of sinefungin when AdoMet is varied at K_M H3$_{1-21}$. Experiments were performed in triplicate and expressed as the mean ± SD. Data was analysed using GraphPad Prism v9.1.

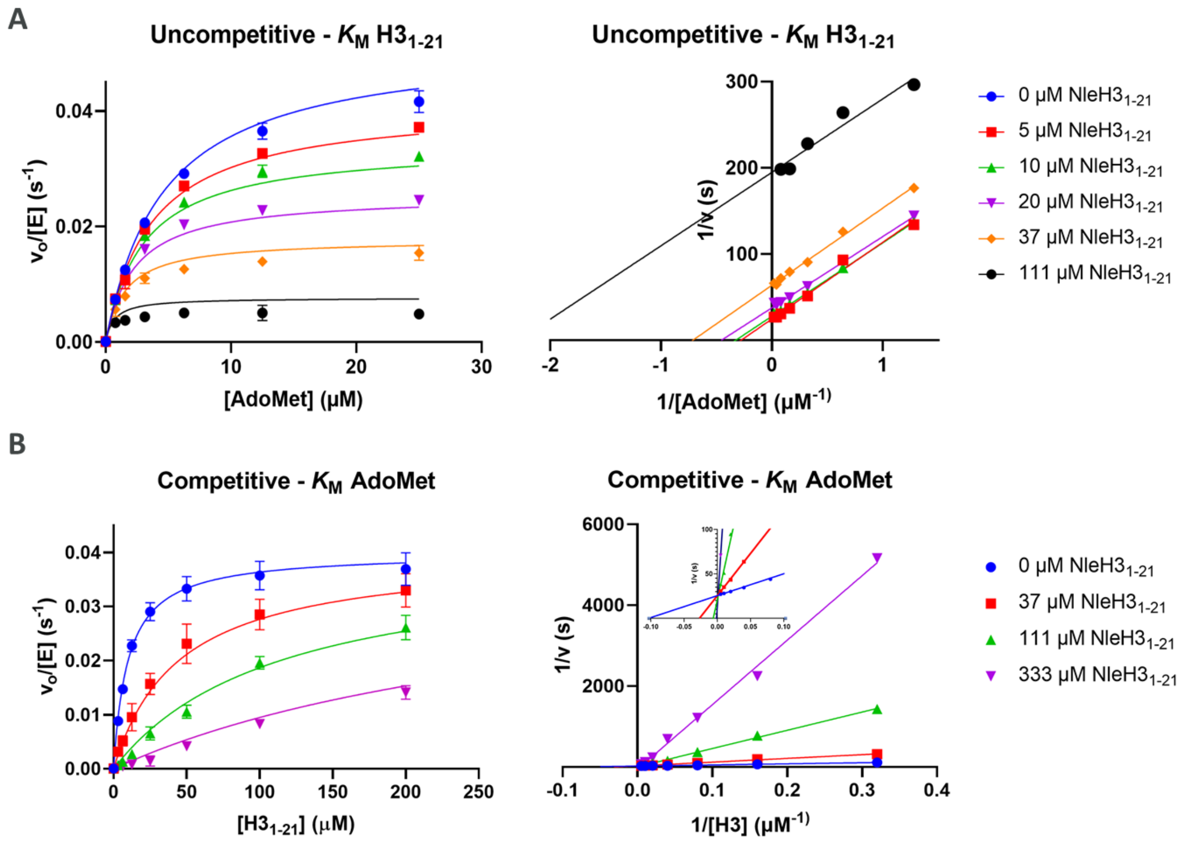

Figure S13. Non-linear and double reciprocal plots of NleH3$_{1-21}$ dead-end inhibitor experiments with SET/WRAD2 at K_M fixed substrate. Double reciprocal fits were generated by calculating $1/k_{cat}$ and K_M/k_{cat} after fitting individual curves to the Michaelis-Menten equation with non-linear regression. (A) shows uncompetitive inhibition of NleH3$_{1-21}$ when AdoMet is varied at K_M H3$_{1-21}$. (B) shows competitive inhibition of NleH3$_{1-21}$ when H3$_{1-21}$ is varied at K_M AdoMet. Experiments were performed in triplicate and expressed as the mean ± SD. Data was analysed using GraphPad Prism v9.1.

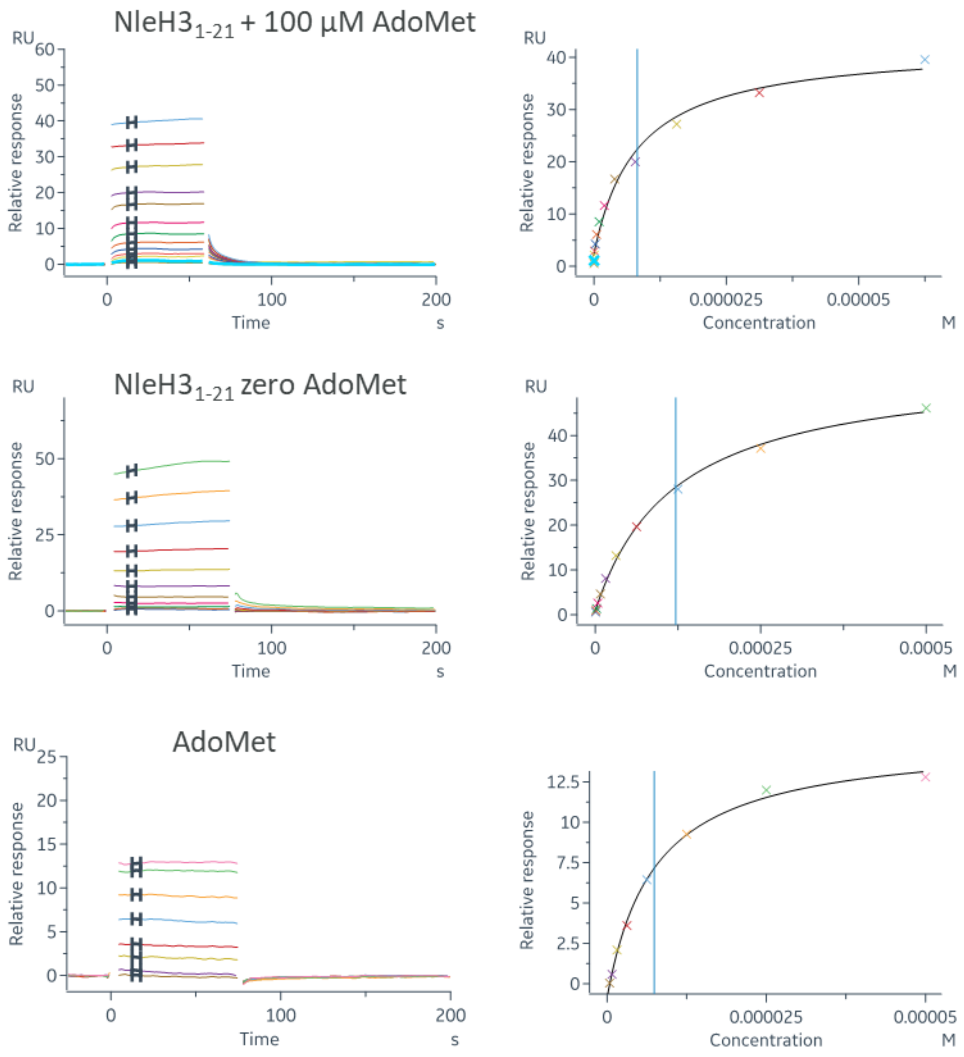

Figure S14. Representative SPR sensorgrams and curve fits. Data fitted using a 1:1 interaction model.

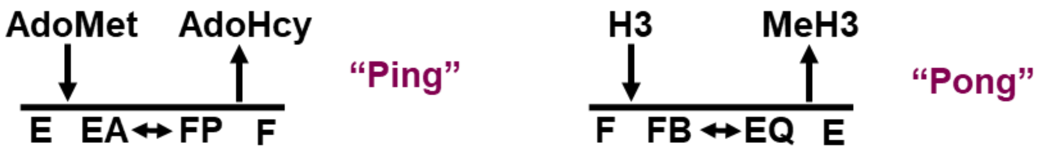

Figure S15. Reaction coordinate of a hypothetical KMT2D Ping-Pong mechanism were a methyl-intermediate of the enzyme is formed. Free enzyme (E) can only bind AdoMet or MeH3 and AdoHcy and H3 can only bind to the covalent intermediate form of the enzyme (F). Therefore, products AdoHcy and MeH3 cannot bind to the same form of the enzyme as their cognate substrates and show competitive inhibition.
